# Diversity of Evoked Astrocyte Ca^2+^ Dynamics Quantified through Experimental Measurements and Mathematical Modeling

**DOI:** 10.3389/fnsys.2017.00079

**Published:** 2017-10-23

**Authors:** Marsa Taheri, Gregory Handy, Alla Borisyuk, John A. White

**Affiliations:** ^1^Department of Bioengineering, University of Utah, Salt Lake City, UT, United States; ^2^Department of Mathematics, University of Utah, Salt Lake City, UT, United States; ^3^Department of Biomedical Engineering, Boston University, Boston, MA, United States

**Keywords:** glia, calcium imaging, GPCR, IP_3_, computational neuroscience

## Abstract

Astrocytes are a major cell type in the mammalian brain. They are not electrically excitable, but generate prominent Ca^2+^ signals related to a wide variety of critical functions. The mechanisms driving these Ca^2+^ events remain incompletely understood. In this study, we integrate Ca^2+^ imaging, quantitative data analysis, and mechanistic computational modeling to study the spatial and temporal heterogeneity of cortical astrocyte Ca^2+^ transients evoked by focal application of ATP in mouse brain slices. Based on experimental results, we tune a single-compartment mathematical model of IP_3_-dependent Ca^2+^ responses in astrocytes and use that model to study response heterogeneity. Using information from the experimental data and the underlying bifurcation structure of our mathematical model, we categorize all astrocyte Ca^2+^ responses into four general types based on their temporal characteristics: Single-Peak, Multi-Peak, Plateau, and Long-Lasting responses. We find that the distribution of experimentally-recorded response types depends on the location within an astrocyte, with somatic responses dominated by Single-Peak (SP) responses and large and small processes generating more Multi-Peak responses. On the other hand, response kinetics differ more between cells and trials than with location within a given astrocyte. We use the computational model to elucidate possible sources of Ca^2+^ response variability: (1) temporal dynamics of IP_3_, and (2) relative flux rates through Ca^2+^ channels and pumps. Our model also predicts the effects of blocking Ca^2+^ channels/pumps; for example, blocking store-operated Ca^2+^ (SOC) channels in the model eliminates Plateau and Long-Lasting responses (consistent with previous experimental observations). Finally, we propose that observed differences in response type distributions between astrocyte somas and processes can be attributed to systematic differences in IP_3_ rise durations and Ca^2+^ flux rates.

## Introduction

Astrocytes are a major glial cell type (Verkhratsky et al., 2012b), playing many key roles in the mammalian brain. Astrocytes are involved in the uptake of neurotransmitters (e.g., glutamate and GABA; Anderson and Swanson, 2000; Zhou and Danbolt, 2013); the release of neuroactive compounds including glutamate, D-Serine, and adenosine 5′-triphosphate (ATP) (Bezzi et al., 1998; Anderson and Swanson, 2000; Haydon, 2001; Newman, 2003; Liu et al., 2004; Wang et al., 2013); regulation of blood flow (Verkhratsky et al., 2012b; Seidel et al., 2015); and K^+^ buffering (Wallraf et al., 2006; Wang et al., 2013; Larsen et al., 2014). Many of these functions are regulated in a Ca^2+^-dependent manner (Kang et al., 1998; Anderson and Swanson, 2000; Haydon, 2001; Wang et al., 2012, 2013; Khakh and Sofroniew, 2015), though the exact mechanisms are still being investigated.

Astrocytes express a variety of functional receptors, mostly metabotropic G-protein-coupled receptors (GPCRs), enabling major communication avenues between neurons and astrocytes. Activation of GPCRs leads to increases in intracellular Ca^2+^ in astrocytes, primarily through the release of inositol (1, 4, 5)-trisphosphate (IP_3_) into the cytosol, which subsequently opens intracellular Ca^2+^ stores (Haydon, 2001). Ca^2+^ increase, in turn, has many effects including, directly or indirectly, driving other transporters and exchangers (e.g., the Na^+^/Ca^2+^ exchanger and Na^+^/K^+^ ATPase pump) (Anderson and Swanson, 2000; Wang et al., 2012) and, possibly, releasing biologically active compounds called gliotransmitters (Bezzi et al., 1998; Haydon, 2001; Liu et al., 2004; Wang et al., 2013). Furthermore, astrocyte Ca^2+^ elevations can propagate through multiple astrocyte subcompartments or multiple astrocytes (Haydon, 2001), enabling inter- and intra-cellular communication. While Ca^2+^ activity is a major method of astrocyte signaling, there is little consensus on its downstream effects and how it may encode information (Pasti et al., 1995; Nedergaard and Verkhratsky, 2012; Evans and Blackwell, 2015). Moreover, the extent of Ca^2+^ response heterogeneity in astrocytes is not well-characterized, though temporal and spatial heterogeneity of Ca^2+^ responses have been addressed by some groups (Verkhratsky and Kettenmann, 1996; Xie et al., 2012; Bonder and McCarthy, 2014; Tang et al., 2015; Jiang et al., 2016).

Computational models of IP_3_-mediated Ca^2+^ responses in astrocytes have been used to investigate Ca^2+^ oscillations (Politi et al., 2006; Ullah et al., 2006; Lavrentovich and Hemkin, 2008; De Pittà et al., 2009; Skupin et al., 2010), influences of astrocytes on neuronal activity (Di Garbo et al., 2007), and the role of intrinsic and extrinsic stochastic events in creating Ca^2+^ response heterogeneity (Toivari et al., 2011). However, many such Ca^2+^ models are closed-cell: they disregard fluctuations in total intracellular Ca^2+^ levels resulting from the activity of plasma membrane channels and pumps. Others models were created based on data from either cell types other than astrocytes or from cultured astrocytes (which are thought to be astrocyte-like cells and not represent *in vivo* astrocytes, Cahoy et al., 2008). Moreover, many models are based on experiments in which agonists were bath-applied to cultured astrocytes, which is far from a physiological stimulation.

Here, we present an integrated, experimental and computational study of ATP-evoked Ca^2+^ transients in astrocytes, with several novel aspects. First, rather than fitting data from bath application of ATP, we use brief focal ATP pulses to astrocytes in acute brain slices that better mimic physiological conditions (Pasti et al., 1995). Second, we use an open-cell mathematical model, accounting for the exchange of Ca^2+^ between the cell and the extracellular space (ECS), to fit the evoked responses. This approach is more realistic (Dupont and Croisier, 2010) and leads to important modeling results. Third, we examine astrocyte response heterogeneity across trials, cells, and subcompartments (i.e., soma and processes) within each cell, and propose a new classification of responses into four types directly motivated by our bifurcation analysis of our mathematical model (Handy et al., 2017): Single-Peak, Multi-Peak, Plateau, and Long-Lasting responses. Experimentally, we find that SP Ca^2+^ response kinetics do not vary consistently between different subcompartments of one astrocyte, but rather vary between cells and trials. In contrast, the frequency of occurrences of observed Ca^2+^ response types varies between astrocyte subcompartments. Using our mathematical model, we explore underlying mechanisms of Ca^2+^ responses and their heterogeneity by varying IP_3_ temporal dynamics and Ca^2+^ channel/pump flux rates. We predict the IP_3_ time course and composition of flux rates that can reproduce the response type distributions observed experimentally for each astrocyte subcompartment, without requiring feedback-induced oscillations in the IP_3_ waveform (Politi et al., 2006). Our model provides a tool to study mechanisms and generate predictions that can be applied to other experimental data.

## Materials and methods

### Ca^2+^ imaging

**Figure 1:**
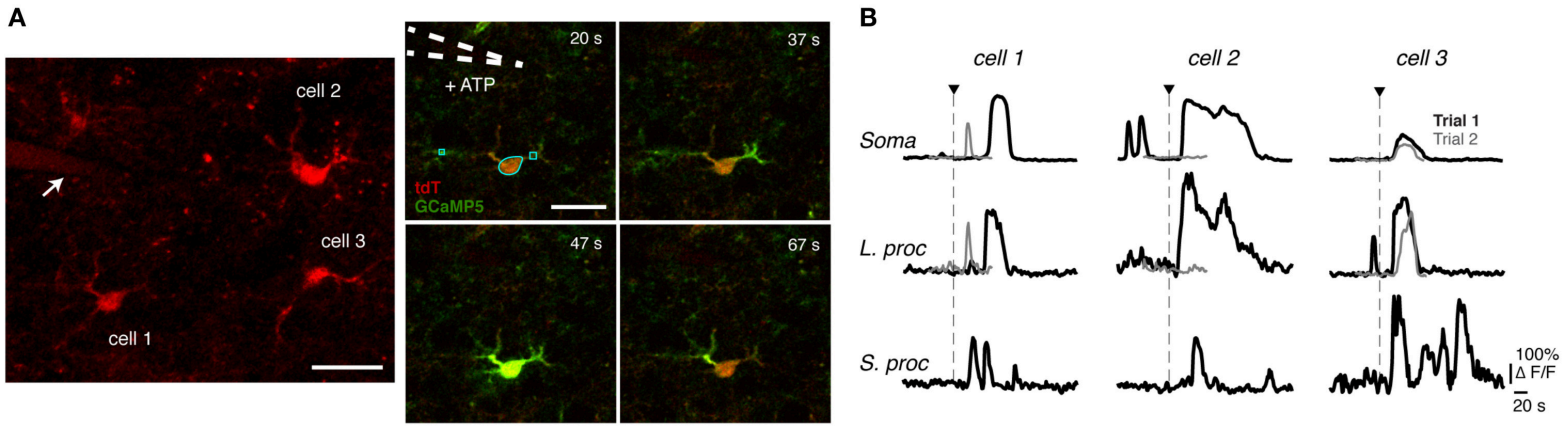
Astrocyte Ca^2+^ imaging and observed response variability. **(A)** (Left) A 12 μm z-stack (centered around the imaging plane) of tdTomato indicating the location of three recorded astrocytes relative to the pipette (shown with arrow), which is also visible since it contains Alexa Fluor 594. The cell numbers correspond to those in **(B)**. (Right) Time series of intracellular Ca^2+^ responses to focal application of ATP (application duration, 63 ms) from a glass pipette (white dotted line) in a mouse acute brain slice. Examples of selected ROIs are shown for the soma, large processes, and small processes (cyan lines in the 20 s frame). These ROIs correspond to the traces of cell 1, trial 1 in **(B)**. Scale bars, 20 μm. **(B)** Example traces of simultaneous responses from different subcompartments of three cells (same cells shown in **A**), in two different ATP application trials.

All procedures were in accordance with the NIH Guide for the Care and Use of Laboratory Animals and approved by the University of Utah Institutional Animal Care and Use Committee. The data were obtained from targeted reporter mice (PC::G5-tdT) crossed with the GFAP-CreER mouse line, and thus express the GCaMP5G genetically-encoded Ca^2+^ indicator in astrocytes (Gee et al., 2014). Cre recombination in GFAP-CreER crosses was induced by a single intraperitoneal injection of tamoxifen in peanut oil (225 mg/kg). All mice were female and were 5–8 weeks old. To extract the brain, the mice were anesthetized in a closed chamber with isoflurane (1.5%) and decapitated. The brains were then rapidly removed and immersed in ice-cold cutting solution that contained 230 mM sucrose, 1 mM KCl, 0.5 mM CaCl_2_, 10 mM MgSO_4_, 26 mM NaHCO_3_, 1.25 mM NaH_2_PO_4_, 0.04 mM Na-Ascorbate, and 10 mM glucose (pH = 7.2–7.4). Coronal slices (400 μm-thick) were cut using a VT1200 Vibratome (Leica Microsystems, Wetzlar, Germany) and transferred to oxygenated artificial cerebrospinal fluid (aCSF) that contained 124 mM NaCl, 2.5 mM KCl, 2 mM CaCl_2_, 2 mM MgSO_4_, 26 mM NaHCO_3_, 1.25 mM NaH_2_PO_4_, 0.004 mM Na-Ascorbate, and 10 mM glucose (pH = 7.27.4; osmolarity = 310 mOsm). Slices were allowed to recover in oxygenated aCSF at room temperature for 1 h before experiments. During the recordings, the slices were placed in a perfusion chamber and superfused with aCSF gassed with 95% O_2_ and 5% CO_2_ at room temperature for the duration of the experiment. To evoke Ca^2+^ responses, 500 μM ATP (Tocris Bioscience, Bristol, UK, catalog no. 3245; similar concentration used by Otsu et al., 2015; Kim et al., 2016) dissolved in aCSF was delivered locally via a glass pipette (10 psi; ranging between 16 and 250 ms with exact values specified in each section or figure) using a Picospritzer III (Parker Instrumentation, Chicago, IL) (see Figure 1A). The pipette also included 5 μM Alexa Fluor 594 so that it could be visualized more readily.

Two-photon imaging was performed using a Prairie two-photon microscope with a mode-locked Ti:Sapphire laser source emitting 140 fs pulses at an 80 MHz repetition rate with a wavelength adjustable from 690 to 1,040 nm (Chameleon Ultra I; Coherent, Santa Clara, CA). We used laser emission wavelengths of 920 nm to excite GCaMP5G or 1,040 nm to excite tdTomato. A 20 × 0.95 NA water-immersion objective was used for all the Ca^2+^ images (Olympus, Tokyo, Japan). Astrocyte Ca^2+^ signaling was recorded at a frame rate of 1 Hz. All imaging was done on cortical astrocytes in the primary somatosensory cortex.

### Data analysis

The two-photon images were processed and analyzed using custom-written MATLAB (2014a, 2015b; MathWorks, Natick, MA) scripts. Each time-lapse image was first processed with a 3 × 3 median filter (using MATLAB's *medfilt2* command). For each pixel, the median fluorescence of 20 frames preceding the agonist application was used to calculate the baseline fluorescence (*F*_0_). Using this baseline, the percent change in fluorescence (100 ^*^ Δ*F/F*_0_) of each pixel was calculated throughout the time-lapse image. Upon displaying the maximum Δ*F/F*_0_ projections, ROIs were manually selected for the soma, large processes, and small processes. Large processes (also known as branches, defined in Khakh and Sofroniew, 2015) were chosen at a distance of ~3–7 μm from the soma perimeter (surrounded with a 2 × 2 μm box). Small processes (also known as branchlets, defined in Khakh and Sofroniew, 2015) were chosen at about 12–20 μm from the soma perimeter (1 × 1 μm box). Finally, the Δ*F/F*_0_ trace for each selected ROI was filtered with an order-3 one-dimensional median filter (using *medfilt1* in MATLAB). The Ca^2+^ response peaks were found using the findpeaks command in MATLAB and were defined as a Δ*F/F*_0_ exceeding *n* standard deviations above the baseline value (where *n* was 7 for the soma and 6 for the processes) and having a value of at least 40%. Only those ROI traces were included in our analyses that responded to the agonist and that had minimal or no spontaneous activity before the agonist application, in order to avoid mistaking spontaneous Ca^2+^ activity for evoked activity.

**Figure 2:**
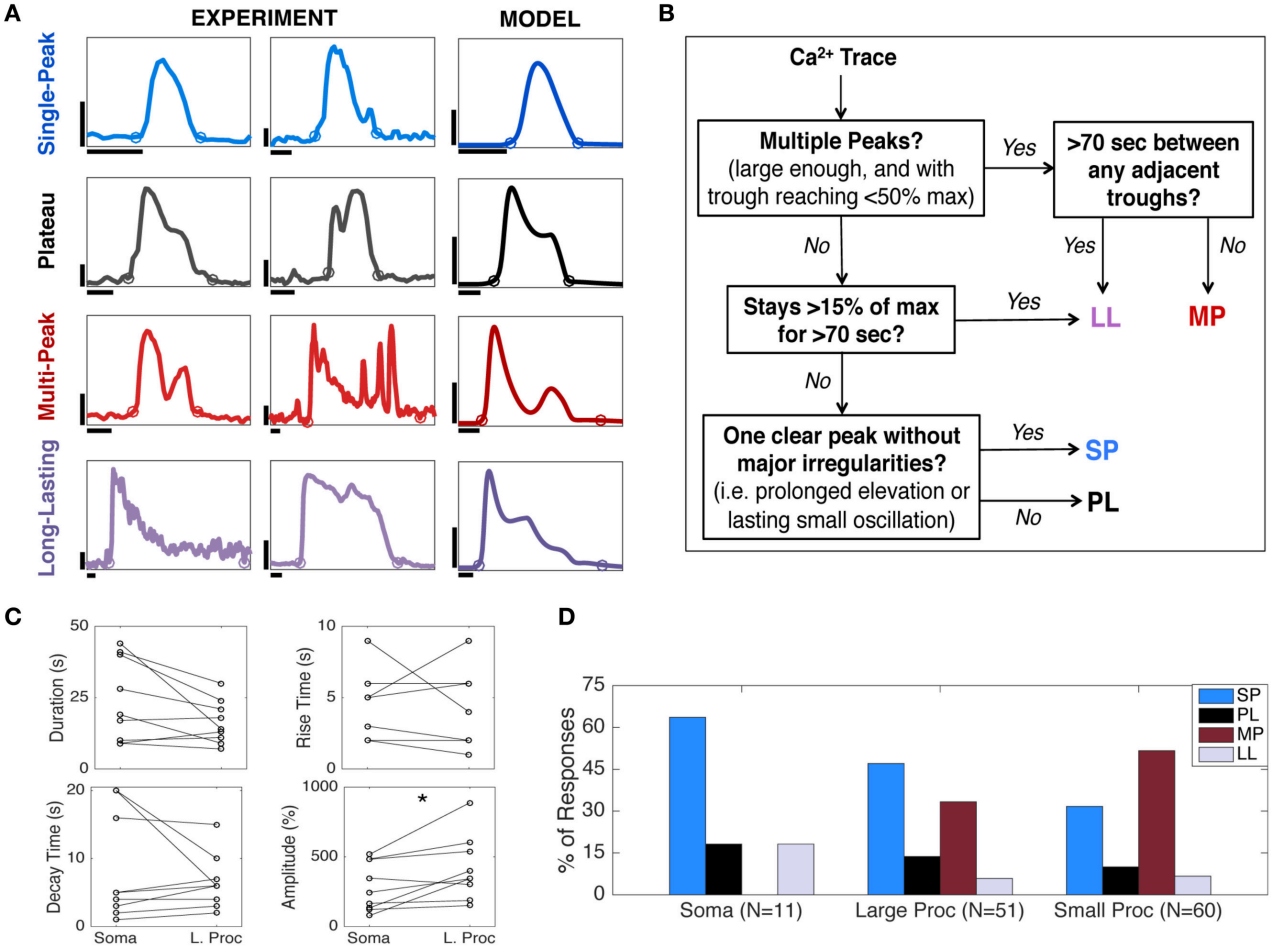
Astrocyte Ca^2+^ response types and kinetics. **(A)** Four categories of astrocyte Ca^2+^ responses observed experimentally (first and second columns) and in the model (third column). Experimental data show astrocyte Ca^2+^ responses to a focal application of ATP. Each of the two experimental columns contain one example of each response type to show the variability within each response category. The model traces are chosen to reproduce responses similar to the first column. For parameter values, see **Figure 4A**. The circles for each trace show the response onset and offset (calculation described in section Materials and Methods). Horizontal scale bars, 10 s; vertical scale bars, 100% ΔF/F for the experiment and 0.5 μM [Ca^2+^]_cyt_ for the model. **(B)** Flow chart summarizing the algorithm for Ca^2+^ response type categorization (details in section Materials and Methods). **(C)** The rise times, decay times, durations, and amplitudes of SP responses in the soma and a large process of the same astrocyte (*N* = 9, stimulus durations 16–250 ms), paired with one another. Only amplitude results were significantly different (^*^p < 0.05; see text), suggesting that there is more variability in SP kinetics between cells and trials than between astrocyte subcompartments. **(D)** The distribution of observed Ca^2+^ response types varies between the somas, large processes, and small processes (*N* = 3 mice, 3 trials per mouse, 3–5 cells per trial, up to 10 ROIs per cell; only responsive cells with minimal spontaneous activity were included here; stimulus durations 30–63 ms).

To calculate Ca^2+^ response durations, the trough before (Trough1) the first response peak and after (Trough2) the last peak were automatically detected. Trough1 (or Trough2) was defined as the last (or first) data point, before (or after) the peak that had a value <3.3 standard deviations above the baseline. The time between the two troughs determined the duration of the experimental Ca^2+^ response. Due to the absence of noise in the mathematical model (described below in section Mathematical Model), simulated responses were determined as the first or last points where the Ca^2+^ concentration reached a value >40% of the baseline. In Figure 2A, the circles on the traces mark the first and last troughs found using this algorithm for both experimentally recorded and simulated traces. The rise and decay times were calculated from the 10–90% points of the rising and falling phases, respectively, of the Ca^2+^ responses.

**Figure 3:**
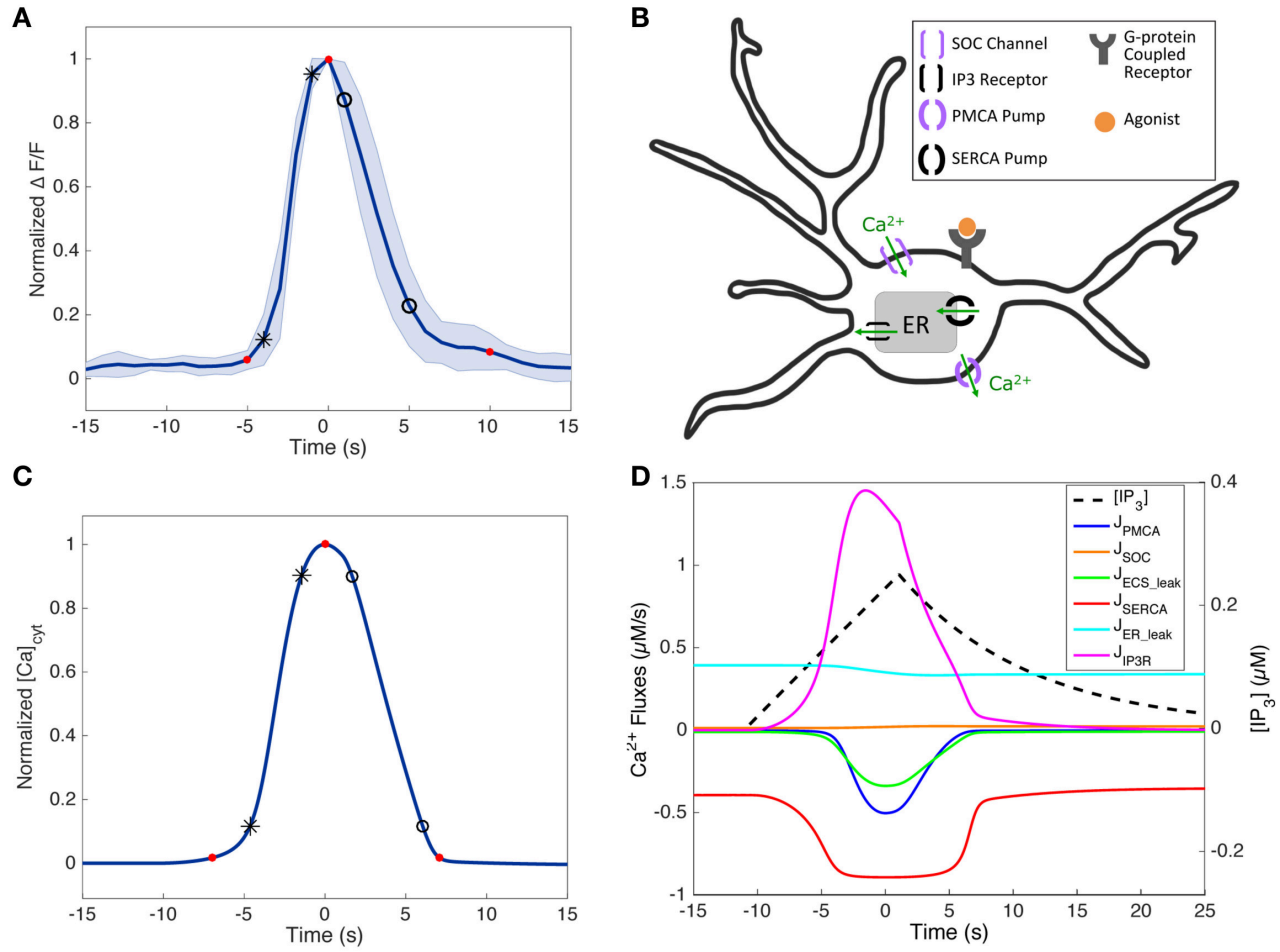
Short-duration SP Ca^2+^ traces in experiments and the model. **(A)** Normalized average short-duration (< 25 s) SP Ca^2+^ traces of the soma (*N* = 8, mean ± standard deviation, stimulus durations 16–250 ms). The response amplitude and latency were ignored by normalizing the amplitudes and aligning the response peaks. **(B)** Simplified schematic of an astrocyte and its major Ca^2+^ components incorporated in the model (except for GPCR dynamics, which is replaced with IP_3_ as the input). Additionally, leak terms between the cytosol and extracellular space (J_ECS_add_) and between the cytosol and the ER (J_ER_leak_) are included in the model. Arrows show the direction of Ca^2+^ flux. The model has only one compartment, tuned to represent data from different astrocyte subcompartments (see section Variability between Astrocyte Subcompartments: Model). **(C)** Normalized SP response in the model, fitted to match the experimental trace in **(A)**. IP_3_ parameters (*A, d*_rise_, *r*_rise_, *d*_dec_): 0.25, 12, 0.002, 40. The experimental and model Ca^2+^ rise times (calculated between the black star markers), decay times (between the black circles), and durations (between the first and last red circles) are as follows: 2.90, 3.90, and 14.7 s (for experiments), and 3.19, 4.38, and 14.05 s (for the model). **(D)** Simulated IP_3_ dynamics and Ca^2+^ fluxes corresponding to **(C)**.

To calculate the experimental normalized average Ca^2+^ trace (Figure 3A), only those SP Ca^2+^ responses were selected that had a short-duration (<25 s) and were from ROIs which had not responded to an agonist application for at least 3 min. The Δ*F/F*_0_ was then normalized such that the maximum peak value for each trace was 1 and the peaks of all traces were aligned (hence, removing any variability in latency from the stimulus time). Finally, the average and standard deviation of the traces were calculated. Similar to Δ*F/F*_0_ traces, the model Ca^2+^ trace (Figure 3B) was normalized and its baseline adjusted such that the trace has a range of 0–1. We used the same duration calculation method for the normalized traces of the experiments and model: the response onset and offset are the first and last points, respectively, that are >20% the baseline value.

### Mathematical model

Because fluorescent signals from GCaMP5G in this animal model are both linear and very fast compared with evoked Ca^2+^ transients (Gee et al., 2014), model results are directly comparable with experimental data, particularly if we compare them independent of response amplitude and latency.

For this comparison, we model astrocyte Ca^2+^ activity in a single compartment, which may represent any one functional subcompartment of an astrocyte. Its parameters can be adjusted to a specific dataset (e.g., the soma or large processes, as we do in **Figure 7A**).

Figure 3B shows a simplified schematic of the Ca^2+^ activity components in our model: activation of a metabotropic G-protein-coupled receptor (GPCR; e.g., P2Y receptors, commonly found on astrocytes) leads to IP_3_ production (We ignore the P2X ionotropic receptor, not found in cortical astrocytes from mice). IP_3_ then binds to IP_3_ receptors (IP_3_Rs) on the endoplasmic reticulum (ER) membrane, which consequently opens IP_3_R channels and allows Ca^2+^ to exit the ER and enter the cytosol. When there is significant depletion of Ca^2+^ from the ER, store-operated Ca^2+^ (SOC) channels are activated, and allow for additional Ca^2+^ to flow from the ECS into the cytosol. Sustained, elevated levels of cytosolic Ca^2+^ inactivate IP_3_Rs, and activate sarco/endoplasmic reticulum Ca^2+^ ATPase (SERCA) pumps and plasma membrane Ca^2+^ ATPase (PMCA) pumps, which then transfer the cytosolic Ca^2+^ back to the ER and ECS, respectively. After the degradation of IP_3_, these pumps and channels return the system to steady state.

Unlike many previous studies on astrocyte Ca^2+^ dynamics (Ullah et al., 2006; De Pittà et al., 2009), our model is an open-cell model in which the total intracellular Ca^2+^ levels can change. Additionally, in this work, we directly control the time course of IP_3_ dynamics, treating IP_3_ as the effective representation of the agonist influence on the cell. This allows us to make predictions as to how the IP_3_ time course affects the resulting Ca^2+^ responses. The general shape of IP_3_ time course is based on previous experimental and modeling work (described below).

Our resulting model consists of a system of three differential equations. Two of those equations model the changes in cytosolic Ca^2+^ concentration, *c*, and total intracellular Ca^2+^ concentration, *c*_*tot*_, as follows:

(1) $$\begin{array}{l} \frac{dc}{dt}=\left[J_{IP3R}\left(c,c_{ER},p\right)+J_{ER\text{\_}leak}\left(c,c_{ER}\right)-J_{SERCA}\left(c\right)\right]\\ +\delta \left[J_{ECS\text{\_}add}\left(c\right)-J_{PMCA}\left(c\right)+J_{SOC}\left(c_{ER}\right)\right], \end{array}$$

(2) $$\begin{array}{l} \frac{dc_{tot}}{dt}=\delta \left[J_{ECS\text{\_}add}\left(c\right)-J_{PMCA}\left(c\right)+J_{SOC}\left(c_{ER}\right)\right], \end{array}$$

and the concentration in the ER is given by *c*_*ER*_ = (*c*_*tot*_ – *c*)γ. We will also denote IP_3_ concentration as *p*. The *J*_*i*_'s in these equations represent fluxes through the pumps and channels that are shown schematically in Figure 3B. *J*_IP3R_ represents the flux from the ER to the cytosol through the IP_3_R channel, *J*_ER_leak_ is the leak between the ER and the cytosol, *J*_SERCA_ captures the flux due to the SERCA pump, *J*_ECS_add_ is the leak between the ECS and the cytosol as well as additional plasma membrane fluxes not explicitly modeled, *J*_PMCA_ represents the PMCA pump, and *J*_SOC_ is the flux through the SOC channels. Further, γ is ratio of the volume of the cytosol to the volume of the ER, and δ is the ratio of membrane transport to ER transport. The remaining differential equation tracks the deactivation rate of the IP_3_R,

(3) $$\begin{array}{l} \frac{dh}{dt}=\frac{h_{\infty }\left(c,p\right)-h}{\tau_{h}\left(c,p\right)}, \end{array}$$

where *h*_∞_ is the equilibrium binding probability and τ_*h*_ is the time constant. We define these quantities more specifically in the following subsections.

#### IP_3_ receptor model

We use the Li-Rinzel IP_3_ receptor (IP_3_R) model to capture the Ca^2+^ dynamics through the IP_3_R channel (Li and Rinzel, 1996). Their model accounts for three binding sites on the receptor: a binding site for activating Ca^2+^, *n*(*c*), deactivating Ca^2+^, *h*(*c,p*), and IP_3_, *m*(*p*). They take the fast variables, the binding of activating Ca^2+^ and IP_3_, to be in quasi-steady state, and they model the slow variable, the binding of deactivating Ca^2+^, explicitly. When open, the flux through the channel is determined by the concentration gradient of Ca^2+^ between the cytosol and the ER. In total, the equations governing this model are

$$\begin{array}{l} J_{IP3R}=v_{IP3R}{m_{\infty }\left(p\right)}^{3}{n_{\infty }\left(c\right)}^{3}h^{3}\left(c_{ER}-c\right), \end{array}$$

where

$$\begin{array}{l} m_{\infty }\left(p\right)=\;\frac{p}{p+d_{1}},n_{\infty }\left(c\right)=\;\frac{c}{c+d_{5}},\\ h_{\infty }\left(c,p\right)=\;\frac{Q_{2}\left(p\right)}{Q_{2}\left(p\right)+c},\tau_{h}\left(c,p\right)=\;\frac{1}{a_{2}\left(Q_{2}\left(p\right)+c\right)},\\ {\;Q}_{2}\left(p\right)=d_{2}\left(\frac{p+d_{1}}{p+d_{3}}\right), \end{array}$$

and the dynamics of h are governed by Equation (3).

The values for the constants can be found in Table 1. This model from Li and Rinzel is a simplification of the one by De Young and Keizer (1992), which was based on data collected on Purkinje neurons. While the structure of the IP_3_ receptor found in astrocytes is most likely very similar to ones found here, the rate constants determining the open probability of receptor are likely to be different. However, little experimental data is available for comparison, and current astrocyte models use constants provided by Li and Rinzel (Ullah et al., 2006; Lavrentovich and Hemkin, 2008; De Pittà et al., 2009). The fast and slow components of this receptor result in excitable behavior. When enough IP_3_ enters the system, Ca^2+^ is released from ER and has a positive feedback onto the receptor, allowing for additional Ca^2+^ release, before the deactivating components of the receptor are bound with Ca^2+^.

**Table 1:** Model parameters.

**Parameter**	**Description**	**Value/Units**
γ	(Cyt vol)/(ER vol)	5.4054
ν_IP3R_	Max IP_3_ Receptor Flux	0.222 s^−1^
ν_ER_leak_	Cytosol to ER leak	0.002 s^−1^
ν_in_	Rate of leak into Cytosol from Plasma Membrane	0.05 μM s^−1^
*k*_out_	Rate of leak out of Cytosol from Plasma Membrane	1.2 s^−1^
ν_SERCA_	Max SERCA Flux	0.9 μM s^−1^
*k*_SERCA_	Half-Saturation for SERCA	0.1 μM
ν_PMCA_	Max PMCA Flux	10 μM s^−1^
*k*_PMCA_	Half-Saturation for PMCA	2.5 μM
ν_SOC_	Max SOC channels Flux	1.57 μM s^−1^
*k*_SOC_	Half-Saturation for SOC channels	90 μM
δ	Scale Factor (ratio of membrane transport to ER transport)	0.2
*d*_1_	Dissociation constant for IP_3_	0.13 μM
*d*_2_	Dissociation constant for Ca^2+^ inhibition	1.049 μM
*d*_3_	Receptor dissociation constant for IP_3_	0.9434 μM
*d*_5_	Ca^2+^ activation constant	0.08234 μM
*a*_2_	Ca^2+^ inhibition constant	0.04 μM-1 s-1
*r*_rise_	Rate of Exponential Growth	[0.002–12] s^−1^
*d*_decay_	Duration of IP_3_ decline	[15–220] s
*d*_rise_	Duration of IP_3_ increase	[1–41] s
*A*	Max amplitude of IP_3_ transient	[0.2–0.9] μM

*γ is from Ullah et al. (2006), k_serca_ and v_serca_ are from De Pittà et al. (2009), ν_in_ is from Lavrentovich and Hemkin (2008), ν_soc_ are found in Croisier et al. (2013), and d_1_, d_2_, d_3_, and d_5_ are from the model developed by De Young and Keizer (1992). Values for ν_IP3R_, ν_leak_, ν_PMCA_, k_out_, k_PMCA_, k_SOC_, δ, and a_2_ were fitted to our data in section Single-Peak Responses and Resulting Model and values for ν_SOC_, ν_PMCA_, ν_SERCA_ were further tuned to our data in section Variability between Astrocyte Subcompartments: Model*.

#### SERCA and PMCA pumps

In the paper by MacLennan et al. (1997), Ca^2+^ flow associated with the SERCA pump was shown to depend sigmoidally on the intracellular Ca^2+^ concentration. We assume that the dependence on Ca^2+^ concentration has a similar form in astrocytes and can be modeled with the following Hill function,

$$\begin{array}{l} J_{SERCA}\left(c\right)=\;v_{SERCA}\frac{c^{1.75}}{c^{1.75}+k_{SERCA}^{1.75}}. \end{array}$$

We fixed the Hill coefficient for this model to be 1.75, as used in Cao et al. (2014), and the default max flow rate (*v*_SERCA_) and the dissociation constant (*k*_SERCA_) were used in De Pittà et al. (2009), and listed in Table 1.

While the SERCA pump is found on the ER, the PMCA pump is found on the plasma membrane and has the ability to pump Ca^2+^ from the cytosol into the ECS. Both pumps require ATP to function, and are believed to have similar dynamics. As a result, we chose to use the following equation for the PMCA pump, as used in Croisier et al. (2013):

$$\begin{array}{l} J_{PMCA}\left(c\right)=\;v_{PMCA}\frac{c^{2}}{c^{2}+k_{PMCA}^{2}}. \end{array}$$

#### SOC channels

Although the molecular mechanism of SOC channels in astrocytes is actively debated, it has been shown that they open when ER Ca^2+^ is depleted, letting Ca^2+^ flow from the ECS into the cytosol (Verkhratsky et al., 2012b). To model this mathematically, we follow Croisier et al. (2013) and set

$$\begin{array}{l} J_{SOC}\left(c_{ER}\right)=\;v_{SOC}\frac{k_{SOC}^{2}}{k_{SOC}^{2}+c_{ER}^{2}}. \end{array}$$

#### Additional membrane fluxes

The model also accounts for an IP_3_R-independent Ca^2+^ leak term between the cytosol and the ER. This term is driven by the concentration gradient between the pools of Ca^2+^ and are a linear approximation of various channels and pumps not modeled explicitly, and is given by the following equation

$$\begin{array}{l} J_{ER\text{\_}leak}\left(c,c_{ER}\right)=\;v_{ER\text{\_}leak}\left(c_{ER}-c\right). \end{array}$$

We also account for additional fluxes across the plasma membrane with the equation

$$\begin{array}{l} J_{ECS\text{\_}add}\left(c\right)=\;v_{in}-k_{out}c, \end{array}$$

where the first term represents the constant leak of Ca^2+^ into the cytosol from the ECS and second term captures additional Ca^2+^ extrusion not explicitly modeled, such as Ca^2+^ extrusion from the Na^+^/Ca^2+^ exchanger (Höfer et al., 2002; Ullah et al., 2006; Keener and Sneyd, 2009; Verkhratsky et al., 2012a).

#### IP_3_ dynamics

As discussed earlier, IP_3_ is produced as a result of GPCR activation. After diffusing in the cytosol and reacting with IP_3_Rs, it diffuses through intercellular gap junctions and/or degrades (Höfer et al., 2002). This biochemical pathway has been studied in astrocytes (Fiacco and McCarthy, 2004; Haydon and Carmignoto, 2006; Petravicz et al., 2008) and included in complex biophysical models (Di Garbo et al., 2007; De Pittà et al., 2009). However, rather than including the production and degradation of IP_3_ explicitly, we opted to treat IP_3_ waveforms as inputs to our model, generated by a simple equation and ignoring possible feedback of Ca^2+^ on IP_3_ (Höfer et al., 2002; Politi et al., 2006), for two reasons. First, this simpler approach made it easier for us to explore how IP_3_ kinetics affect the shape of Ca^2+^ events in the absence of feedback. Second, because IP_3_ production and degradation have not been measured in astrocytes, treating IP_3_ as an input reduces the number of free parameters in the model substantially. Our simple model makes the following assumptions: following a pulse of ATP, IP_3_ exponentially saturates to a level denoted as s_∞_, and then exponentially decays back to steady state:

(4) $$\begin{array}{l} p\left(t\right)=\{\begin{array}{c} 0\;t<t^{*}\\ s_{\infty }\;\cdot \;(1-e^{-r_{rise}\left(t-t^{*}\right)})\;t^{*}\leq t<t^{*}+d_{rise}\\ \;A\;\cdot \;e^{-r_{dec}\cdot \left(t-\left[t^{*}+d_{rise}\right]\right)}\;t^{*}+d_{rise}\leq t \end{array} \end{array}$$

where

$$\begin{array}{l} s_{\infty }=\frac{A}{1-e^{-r_{rise}\cdot d_{rise}}},r_{dec}=-\frac{1}{d_{dec}}\log\left(\frac{0.005}{A}\right), \end{array}$$

t^*^ is the time of stimulus, *A* is the max amplitude, *r*_rise_ and *r*_dec_ are the rate of rise and decay respectively, and *d*_rise_ and *d*_dec_ are the durations (0–100% and vice-versa) of the rising and decaying phases, respectively.

To determine a reasonable range of the IP_3_ parameters *A, r*_rise_, *d*_dec_, and *d*_rise_ for use in our simulations, we examined and compared data from previous experimental (Pasti et al., 1995; Tanimura et al., 2009; Nezu et al., 2010) and modeling (De Pittà et al., 2009) studies. Tanimura et al. (2009) and Nezu et al. (2010) had imaged IP_3_ dynamics during ATP-induced (bath applied) Ca^2+^ responses of COS-7 and HSY-EA1 cells. While they found differences in peak IP_3_ concentrations within one cell type, between the two cell types, and with different ATP concentrations (Tanimura et al., 2009; Nezu et al., 2010), we used their results and IP_3_ traces to estimate a range of IP_3_ amplitudes, rise durations, and decay durations. We also ran simulations using the detailed GPCR model developed by De Pittà et al. (2009) to generate IP_3_ dynamics and Ca^2+^ responses to different glutamate concentrations applied for short durations (<5 s). We compared these results with results from local, brief (<100 ms) glutamate applications in cultured astrocytes by Pasti et al. (1995) and accounted for the observed discrepancies by adjusting model parameters, to obtain a reasonable range of IP_3_ amplitudes, total durations, rise durations, and decay durations. The complete set of IP_3_ parameters we chose to use in our model is as follows: A = (0.2, 0.375, 0.55, 0.725, 0.9), *r*_rise_ = (0.002, 0.04, 0.07, 0.09, 0.12, 0.15, 0.3, 0.44, 0.8, 1, 1.6, 12), *d*_dec_ = (15, 56, 97, 138, 179, 220), and *d*_rise_ = (1, 11, 21, 31, 41) (resulting in a total of 600 IP_3_ traces; only a subset of *r*_rise_ value were used for each *d*_rise_-value, in order to avoid creating repetitions in the IP_3_ time courses). All 600 IP_3_ traces were used throughout this paper, unless otherwise noted in the figure captions and text.

### Parameter fitting

While the mechanisms behind the Ca^2+^ fluxes, and hence the mathematical form of these fluxes, are similar between cell types, it is known that the specific dynamics and time scales of these channels can vary. Further, many of these channels and pumps have not been investigated in astrocytes with sufficient detail to capture specific parameter values. As a result, we fitted several parameters (*v*_ip3r_, *v*_leak_, *v*_pmca_, *k*_out_, *k*_pmca_, *k*_soc_, δ, *a*_2_) of our model in order to match the kinetics of an average, short-duration (<25 s) experimental Ca^2+^ transient (Figure 3A). We first established a reasonable IP_3_ transient to act as a driving force for short-duration Ca^2+^ transients, and then fitted parameters accordingly by hand. In addition to fitting the experimental data, we also required the model to have a realistic resting astrocyte Ca^2+^ concentration in the cytosol (~0.1 μM) and the ER (~200 μM) (Verkhratsky and Butt, 2007). All fitted parameters are similar in magnitudes as those found in the literature and the complete list of parameter values can be found in Table 1. The fitted, normalized model simulation can be seen in Figure 3B.

### Monte Carlo simulations

To account for experimental variability between astrocyte subcompartments (soma, small, and large processes; Figure 2D), we considered a broader parameter space than what is found in Table 1. We ran 30 simulations for each IP_3_ transient (each IP_3_ parameter set in Table 1), choosing *v*_pmca_, *v*_serca_, and *v*_soc_ from a uniform distribution centered at the default value found in Table 1, with a maximum set at 150% of this value and minimum set at 50%. The system was first allowed to equilibrate to steady state with these new parameters, and then the IP_3_ stimulus was applied and Ca^2+^ response recorded. Once this data set was created, we separated the three dimensional parameter space into 27 subspaces (based on the values of *v*_pmca_, *v*_serca_, and *v*_soc_), and examined the distribution of Ca^2+^ response types in each subspace. We also examined these distributions while limiting the ranges of IP_3_ *d*_rise_ parameters, which were divided into increasingly larger ranges: *d*_rise_-values from 1 to 11, 1 to 21, 1 to 31, and 1 to 41 s (the full range of *d*_rise_, as listed in Table 1). After examining the response type distributions, **Figure 7** was created by choosing the parameter subspace, or subspaces, that best matched the experimental data in Figure 2D.

### Categorization of Ca^2+^ response types

After examining the astrocyte literature, our experimental data, and the model simulations, we categorized all Ca^2+^ responses into four major categories based on their duration and shape: Single-Peak (SP), Plateau (PL), Multi-Peak (MP), and Long-Lasting (LL). A general definition for each response type is as follows (with corresponding examples and flowchart in Figures 2A,B):

#### Multi-peak

A signal that has more than one peak in succession (for experimental data, ≤16 s gap between each consecutive response), with at least one trough reaching <50% of the maximum adjacent peak height. Only those peaks are considered that have heights >5% of the adjacent peak height.

#### Long-lasting

A signal that stays elevated continuously, without returning close to baseline (i.e., <15% of the maximum adjacent height) or having additional peaks (with troughs reaching <50% of the maximum adjacent height) for more than 70 s. When there are multiple peaks, if the signal remains elevated for >70 s between any two adjacent troughs, it will be considered a LL response; otherwise, it will be a MP response.

#### Single-peak

A signal with one clear peak, without any subsequent major oscillations or bumps. A major oscillation/bump is one with a sufficiently large height (>5% of the adjacent peak height) or sufficiently long duration (lasting >50% of the main peak's duration).

#### Plateau

A signal with one main peak and subsequent bump or oscillation that either has a sufficiently long duration (>50% of the main peak's duration) or is elevated with its troughs >50% of the peak heights.

In our mathematical model, when changing the IP_3_ time course, the simulated Ca^2+^ responses transition between response types as a continuum. As a result, to automatically determine the cytosolic Ca^2+^ response types of the mathematical model simulations, we developed an extensive MATLAB script to implement the classification procedure described above (more details and the MATLAB scripts can be found in the ModelDB database; Hines et al., 2004; http://senselab.med.yale.edu/modeldb/default.asp; Model no. 189344). It is also worth noting that Ca^2+^ responses that had amplitudes too small to be detected experimentally (<0.4 μM) or had amplitudes too high to be biologically reasonable (>3.5 μM) were not included in our analyses.

For experimental traces, Ca^2+^ response types were determined using a similar algorithm; however, due to inherent noise in experimental signals, the algorithm was manually implemented rather than using the MATLAB script. Moreover, the following step was modified to avoid mistaking inherent experimental noise for additional peaks in the Ca^2+^ traces: in the case of a second peak or oscillation, to determine if the peak was large enough to be a MP response, we checked if its height was >15% of the adjacent peak height (rather than >5% as for the model traces).

### Statistics

The paired sample *t*-test was used to compare SP kinetics between the soma and large processes. Pearson's chi-square test was used to compare the distributions of experimental Ca^2+^ response types recorded from the soma, large processes, and small processes. Fisher's exact 2-tail test was used to compare the frequencies of specific Ca^2+^ response types recorded in these subcompartments. The Kolmogorov-Smirnov test was used to compare the distribution of Ca^2+^ durations observed experimentally and generated in the model using different subsets of IP_3_ transient parameters.

## Results

### Experimentally-observed variability in evoked Ca^2+^ responses

We applied brief (16–250 ms) local pulses of ATP and examined Ca^2+^ responses in three subcompartments of each imaged astrocyte: the soma, large processes, and small processes (Figure 1A; regions of interest are marked for one cell at *t* = 20 s). In Figure 1B, we plot Ca^2+^ traces vs. time for the three cells, with cell 1 being the astrocyte from the time-lapse image in Figure 1A. In such data, we observed variability among simultaneously recorded responses of different cells, simultaneous responses of different subcompartments within a given cell, and between trials in a given subcompartment. In the first trial (black traces), simultaneous responses of three cells differed substantially, and they each displayed differences among their subcompartments (soma, large processes, and small processes). In the second trial (gray traces; with 5 min of rest between trials and the same agonist concentration, agonist application duration, and pipette location as in trial 1), cell 1's soma and large process (same process as in trial 1) responded with a smaller amplitude, duration, and latency. On the other hand, the soma and the same large process of cell 2 failed to respond to the second stimulus. In contrast, cell 3 responded consistently between the two trials. Such variability between trials, subcompartments, and cells was not uncommon in our experiments. Even when agonists are bath applied and the spatial variability of the agonist concentration is smaller than with our ATP pulse experiments, responses in different cells or astrocyte subcompartments vary greatly in their shapes and durations (e.g., Xie et al., 2012). Our goal in this paper is to characterize these different forms of response variability (summarized in Table 2) in more detail, to develop a mathematical model that generates the variety of observed Ca^2+^ responses (i.e., with a variety of temporal features, similar to those seen experimentally), and to use the model to examine the sources of these forms of response variability (Table 2), particularly the spatial variability among different astrocyte subcompartments.

**Table 2:** Experimentally observed variability in Ca^2+^ dynamics.

**Forms of Ca^2+^ response variability**	**Likely source of variability**	
	IP_3_ dynamics^*^	**Ca^2+^ channels/pumps**
Among different astrocytes	Yes	Yes
Among different subcompartments of one astrocyte	Yes	Yes
From trial to trial (same ROI and agonist amount)	Yes	No

* *The differences in IP_3_ dynamics may be a consequence of either differences in GPCR expression or functional properties, spatial diffusion of IP_3_ and consequent interactions, or stochasticity in the production and degradation of IP_3_, downstream of GPCR activation*.

In order to quantify diversity in astrocyte Ca^2+^ signaling, we divided all Ca^2+^ responses into four main categories according to their shape and duration: Single-Peak (SP), Plateau (PL), Multi-Peak (MP), and Long-Lasting (LL). This response type categorization is based on our observed experimental data, the astrocyte literature (Verkhratsky and Kettenmann, 1996; Xie et al., 2012; Bonder and McCarthy, 2014), and the mathematical structures underlying our model dynamics, described in section Model Verification and in Handy et al. (2017). In Figure 2A (first and second columns), we show example traces of cytosolic Ca^2+^ elevations elicited by 30–63 ms applications of ATP. For each response type, two examples of experimental recordings are shown in order to illustrate the variability of observed Ca^2+^ responses within each response category. In the third column of Figure 2A, we show a stereotypical response for each class, generated by our model, chosen to match the experimental responses in the first column (for model details see section Model Verification).

Figure 2B shows a flow chart summarizing the categorization algorithm (see section Materials and Methods for additional details on response type definitions). A similar classification of responses was proposed by Xie et al. (2012), where astrocyte Ca^2+^ responses to agonist-bath applications (with durations of tens of seconds) were categorized into three classes based only on the response shape. We also categorized responses based on duration, particularly separating those responses that lasted 70 s or more (described in Khakh and Sofroniew, 2015) into a separate category. We altered some definitions of the three response types proposed by Xie et al. (2012) in order to incorporate this fourth response type, as well as to describe the variability not only in our experimental data, but also in our model simulations using the same algorithm.

### Forms of Ca^2+^ response variability and their sources

Having established the presence of a variety of responses in the data, we next sought mechanisms that underlie the observed evoked Ca^2+^ response variability under different contexts (summarized in Table 2). In subsequent sections, these mechanisms will be explored in detail in our mathematical model.

#### Trial to trial and cell to cell variability

Our results show that the same recording site (region of interest, ROI) can respond differently to identical agonist pulses in different trials (cf. the black and gray traces in Figure 1B). For two reasons, we believe that variability in the spatiotemporal synthesis and degradation of IP_3_ are the main contributors to trial to trial variability. First, it seems unlikely that GPCR and Ca^2+^ channel/pump properties change on the short time scale of our experiments (<20 min). Second, IP_3_ uncaging experiments provide direct evidence that IP_3_ is the sole source of trial to trial variability. Fiacco and McCarthy (2004) found that astrocyte Ca^2+^ response kinetics to multiple IP_3_ uncaging trials were consistent in any one cell, suggesting that trial to trial variability in agonist application experiments stems mainly from factors upstream of the IP_3_ waveform.

Interestingly, Fiacco and McCarthy (2004) did observe variability in response duration from cell to cell in their IP_3_ uncaging experiments. This finding supports the hypothesis that different cells are likely to have inherently diverse properties downstream of IP_3_ dynamics. Compatible with this hypothesis, cell to cell variability is also seen in our data (Figure 1B) and in response to bath-applied agonists (e.g., Xie et al., 2012). We speculate that cell to cell variability is dominated by different distributions and properties of Ca^2+^ channels and pumps, in addition to differences in GPCR expression levels (and subsequent differences in IP_3_ kinetics). We explore this idea in the simulations described later.

#### Variability between astrocyte subcompartments

Previous studies (e.g., Tang et al., 2015) have reported differences in Ca^2+^ kinetics between the astrocyte soma and processes in response to neural stimulation. Using ATP application, we investigated whether the kinetics of SP responses (the most commonly observed response type in our experiments, as described below) varied between the soma and large processes of individual astrocytes. To control for trial to trial and cell to cell variability, we examined pairs of somas and large processes of the same astrocyte that responded to the same trial of ATP application with a SP response (*N* = 9). While we found that the Ca^2+^ amplitude is greater in large processes than in somas (paired *t*-test, *p* = 0.023), we found no significant differences between the SP durations (*p* = 0.059), rise times (*p* = 0.586), or decay times (*p* = 0.367) (Figure 2C). The difference in amplitudes could be a result of differences in ROI sizes and consequent ΔF/F_0_ calculations, which involve averaging over the ROI area, as opposed to intrinsic differences in response kinetics.

While we did not find significant differences between the paired SP response kinetics of the somas and large processes, we did note differences in the likelihood of observing certain types of responses in each of these subcompartments. In Figure 2D, we plotted the distribution of response types observed in the somas, large processes, and small processes in our experiments over nine trials (three mice). Our results indicate that SP responses are the most common response type in the soma, while MP responses are rarest. However, the finer the astrocyte processes, the more likely they are to exhibit MP responses instead of SP transients (Fisher's exact 2-tail test, between the MP responses of somas and large processes *p* = 0.026, and of the somas and small processes *p* = 0.0016). Further, PL and LL responses were observed at a lower rate in all three subcompartments. The following are the percentages of observed response types (in order, SP, PL, MP, LL) in each astrocyte subcompartment: 63.64, 18.18, 0.0, 18.18% (somas); 47.06, 13.73, 33.33, 5.88% (large processes); 31.67, 10.0, 51.67, 6.67% (small processes). In comparing the overall response type distributions, we found the largest differences to be between the distributions of the somas and small processes (Pearson's chi-squared test, *p* = 0.016).

The variability in response type distributions between subcompartments could be the result of differences in both the IP_3_ dynamics and the functional properties of Ca^2+^ channels and pumps. As above, the differences in IP_3_ dynamics within different astrocyte subcompartments could arise from differences in GPCR expression levels or functional properties, differences in agonist binding probability or IP_3_ diffusion within or between cells (due to differences in subcompartment size and shape), or differences in regulatory mechanisms downstream of GPCR activation. Moreover, differences in functional properties of Ca^2+^ channels and pumps in different astrocyte subcompartments could also arise from differences in subcompartment size and shape, differences in expression levels and spatial distributions of these Ca^2+^ components, or simply different activity levels of these components.

To summarize, we suggest that the mechanisms behind the observed variability in all three cases (Table 2) stem mainly from differences in one or both of the following: (1) IP_3_ dynamics, (2) Ca^2+^ fluxes through various Ca^2+^ handling mechanisms (e.g., SOC channels). We will next examine the plausibility and consequences of each of these mechanisms in our mathematical model (some other potential sources of variability, e.g., the volume ratio of the cytosol to the ER, have also been examined in our model (Handy et al., 2017) but were found to not have a major effect on Ca^2+^ responses).

### Model verification

Our single-compartment model includes the Ca^2+^ handling mechanisms shown in Figure 3B, with IP_3_ (rather than the GPCR agonist) as the direct input. It consists of three ordinary differential equations describing changes in cytosolic Ca^2+^ levels, total intracellular Ca^2+^ levels, and the inactivation variable of the IP_3_ receptor (IP_3_R). See section Materials and Methods for details and Handy et al. (2017) for a general bifurcation analysis. Here, we fit the model to experimental data in order to understand the factors that contribute to response variability in response to brief pulses of ATP.

#### Single-peak responses and resulting model

To verify our model of astrocyte Ca^2+^ dynamics, we first ensured that our model matches experimentally observed Ca^2+^ kinetics during a typical SP Ca^2+^ response, which is the most common astrocyte Ca^2+^ response type when stimulated with a brief agonist pulse (Figure 2D). We examined experimental short-duration (<25 s) SP Ca^2+^ responses and found that the average of such a response for the somas (*N* = 8; Figure 3A) was similar in kinetics to that of the processes (*N* = 13, data not shown), in agreement with the results discussed in Figure 2C. Given this similarity, and the fact that the somatic responses were less variable and noisy, we chose to fit our model parameters to the average trace from the soma (Figure 3A).

Because short-duration SP responses are the briefest observed astrocyte Ca^2+^ responses, we used an IP_3_ input within the lower end of the parameter range in Table 1 (specific values in Figure 3 caption) to generate a SP response in the model. The fitted SP simulation, along with the corresponding IP_3_ dynamics and Ca^2+^ fluxes driving this response, is shown in Figures 3C,D. Before the IP_3_ stimulus, the simulated system is at steady-state, with the Ca^2+^ fluxes governed by the ER leak and SERCA pump balanced. When IP_3_ is released into the cytosol, the IP_3_R becomes activated and Ca^2+^ flows from the ER into the cytosol. Depletion of ER Ca^2+^ causes SOC channel activation, though this flux remains small for the duration of this response. The increase in cytosolic Ca^2+^ also quickly activates the SERCA pumps, allowing for Ca^2+^ to be pumped back into the ER. As the cytosolic Ca^2+^ concentration continues to rise, the PMCA pumps also become activated and some Ca^2+^ is lost to the ECS. Additional Ca^2+^ is also released into the ECS due to the ECS_add term. Moreover, before cytosolic IP_3_ begins to degrade, the Ca^2+^ flux through the IP_3_R decreases as a result of negative feedback from elevated levels of cytosolic Ca^2+^. Together, the SERCA pump, PMCA pump, ECS_add, and the deactivation of the IP_3_R are able to slow the net change of Ca^2+^ in the cytosol. As IP_3_ is degraded and removed from the system, the IP_3_R begins to deactivate more rapidly, and the pumps are able to return the system to equilibrium. Even when the Ca^2+^ response measured in the cytosol has returned to baseline levels, the pumps and channels continue to work at a low level to restore the system back to pre-stimulus equilibrium. In the model, the process of refilling the ER to pre-stimulus levels is on the order of ~10 min.

#### Heterogeneity of model Ca^2+^ responses

**Figure 4:**
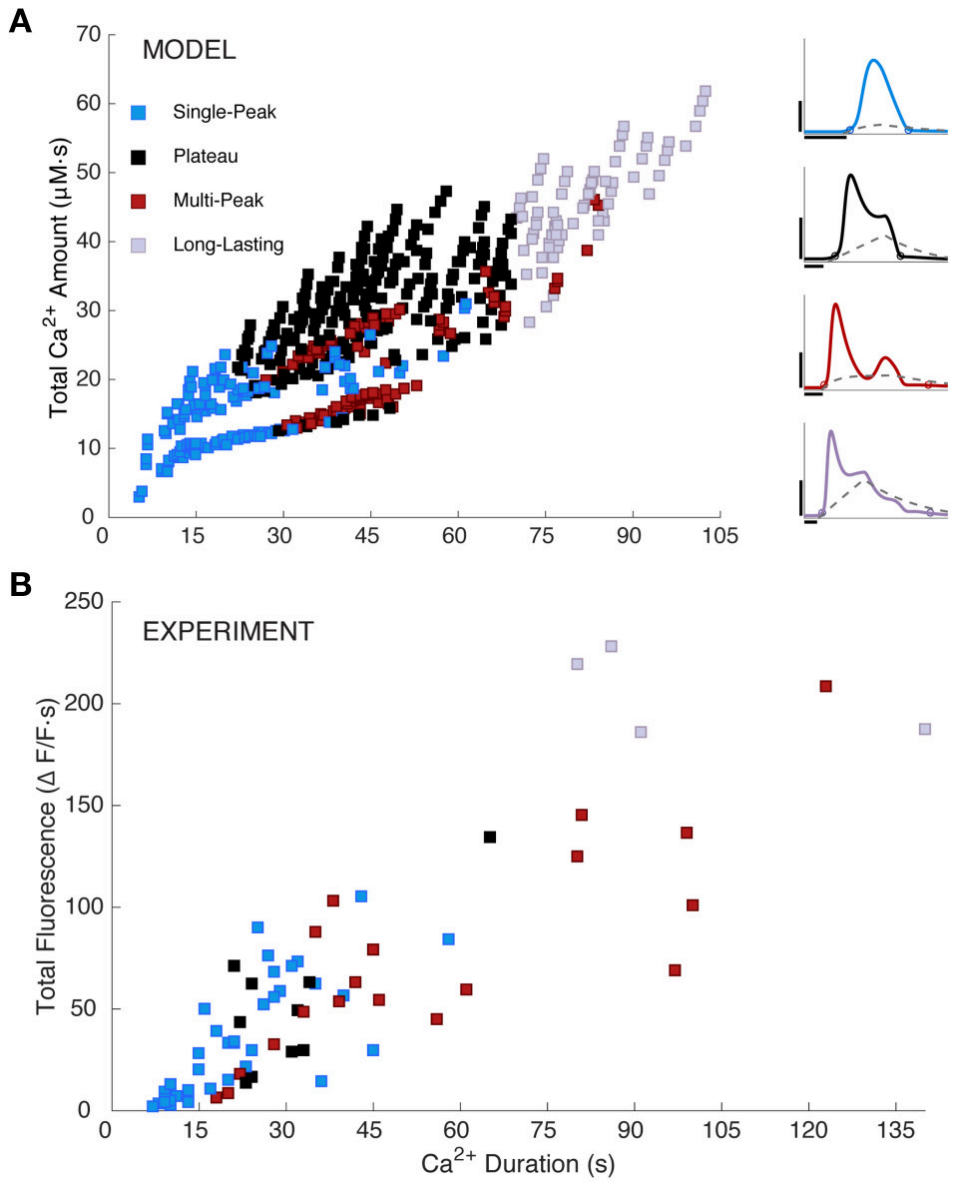
Simulated and measured Ca^2+^ responses. **(A)** Simulated total Ca^2+^ amount (the area under the [Ca^2+^]_cyt_ curve) vs. Ca^2+^ duration as defined in section Materials and Methods. Model responses were generated by choosing IP_3_ parameter sets as specified in section Materials and Methods and Table 1. Response types are indicated as shown. The right panel repeats example Ca^2+^ traces from Figure 2A, column 3 with their respective IP_3_ time courses added. The IP_3_ parameters are as follows (in order, *A, d*_rise_, *r*_rise_, *d*_dec_): 0.2, 10, 0.2, 90 (SP); 0.375, 34, 0.002, 110 (PL); 0.26, 41, 0.15, 200 (MP); 0.6, 39, 0.002, 220 (LL). Horizontal scale bars, 10 s; vertical scale bars, 0.5 μM. **(B)** Experimentally measured Ca^2+^ responses (y-axis shows area under the ΔF/F trace; *N* = 19 somas and 52 large processes from a total of 3 mice, 3–6 trials per mouse, 2–5 cells per trial; 16–250 ms stimulus durations) are similar to modeled responses, but more sparsely and non-uniformly distributed. One outlier point was omitted from **(B)**, at a duration and total fluorescence of about 363.

As discussed in section Forms of Ca^2+^ Response Variability and their Sources, IP_3_ stochasticity is likely a major source of all three forms of variability in evoked Ca^2+^ responses considered in this manuscript (see Table 2). To explore this issue, we changed IP_3_ parameters (*A, d*_rise_, *r*_rise_, and *r*_dec_; linear spacing) over a biologically plausible range of values (see section Materials and Methods and Table 1 for details), while other model parameters were fixed at the default values. We found that this range of IP_3_ kinetics was sufficient to reproduce our experimentally-observed Ca^2+^ responses, compatible with our bifurcation-based classification scheme (for details on how each response type arises from a given IP_3_ waveform, refer to Handy et al., 2017). Examples of the response types are shown in Figure 4A on the right (solid lines, repeated from Figure 2A), along with the underlying IP_3_ waveforms (dashed lines).

Furthermore, Figure 4A shows a color-coded scatter plot of the four response types generated by this parameter search, plotted vs. response duration and the total area under the simulated Ca^2+^ response curve (i.e., the total Ca^2+^ amount). For comparison, in Figure 4B we show a similar scatter plot for the experimental data (*N* = 71). Model and experimental responses are qualitatively similar, dominated by SP responses for durations <22 s; MP or LL responses for durations >70 s; and a mix of SP, MP, and PL responses for intermediate durations. For both the model and experiments, duration and total area under the curve (i.e., total Ca^2+^ amount for model, and total fluorescence for experiments) are positively correlated. Additionally, the range of Ca^2+^ response durations between simulations and experiments are similar. These similarities suggest that the range of our model parameters and selected IP_3_ kinetics are biologically plausible for evoked, IP_3_-dependent astrocyte Ca^2+^ activity. However, experimental Ca^2+^ responses are more sparsely and non-uniformly distributed than model Ca^2+^ responses, which we explore next.

### IP_3_ dynamics as a source of response variability in the model

Although the range of Ca^2+^ response durations is similar in both model and data (Figure 4), the distributions of response durations are significantly different (Supplementary Figure 1, Kolmogorov-Smirnov test, *p* = 8e-6), with longer PL and LL responses overrepresented in simulations. Hypothesizing that IP_3_ kinetics may influence the distributions of model response durations, we explored the effects of restricting the IP_3_ waveform, with the goal of matching the distribution of experimental response durations without eliminating altogether the long-duration responses of Figure 4A. Of the many manipulations we attempted (i.e., limiting IP_3_ total duration, decay duration, rise duration, and amplitude), we only succeeded in this goal by limiting the IP_3_ rise duration (*d*_*rise*_) from Equation (4) to <22 s (Kolmogorov-Smirnov test, *p* = 0.2, max duration = 83 s, Supplementary Figure 1). Thus, the model suggests that shorter IP_3_ rise durations are more common in the experimental data set than the full range of model IP_3_ parameters originally considered.

**Figure 5:**
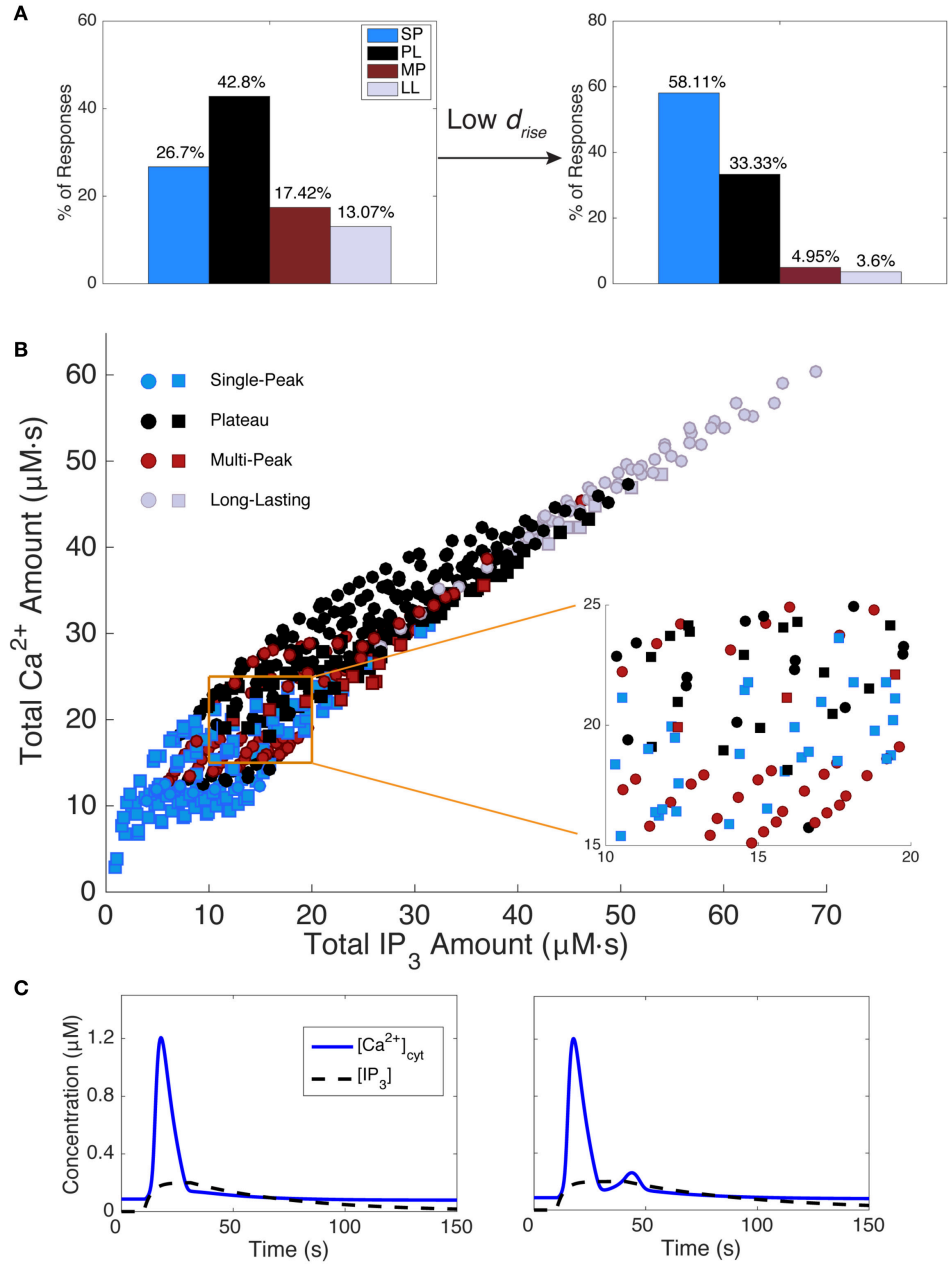
Effect of IP_3_ kinetics on Ca^2+^ responses. **(A)** The left histogram shows the distribution of model response types while scanning IP_3_ kinetics over the full parameter range from Table 1. The right histogram plots the response type distribution for shorter IP_3_ rise durations (*d*_rise_ < 22 s). Shorter IP_3_ rise durations tend to decrease the occurrence of mostly MP and LL responses, with SP being the major response type, while longer IP_3_ rise durations decrease the percentage of SP responses, but increase other response types. **(B)** The total IP_3_ amount correlates with the total Ca^2+^ amount. However, in most regimes, within a small range of IP_3_ (and Ca^2+^) amounts, a mixture of Ca^2+^ response types are generated. An example of such a regime is shown in the zoomed section. Squares indicate responses generated with *d*_rise_ < 22 s (corresponding to data set in the right histogram in **(A)** and circles indicate responses generated with all other *d*_rise_ values from Table 1. **(C)** Two example Ca^2+^ responses are shown with their underlying IP_3_ dynamics. As seen, a small change in IP_3_ kinetics is sufficient to change the Ca^2+^ response type from SP to MP, while the total IP_3_ amount remains roughly the same (15.28 and 15.17 μM, respectively; Ca^2+^ amounts are 13.86 and 15.56 μM, respectively). IP_3_ parameters (*A, d*_rise_, *r*_rise_, *d*_dec_): 0.2, 21, 0.3, 220 (SP), 0.2, 31, 0.3, 179 (MP).

Because response durations and types are clearly correlated (Figure 4), we might expect that restricting *d*_*rise*_ will alter the distributions of response types as well. Figure 5A shows the distribution of modeled response types for the default range of IP_3_ parameters (left panel) and for restricted values of rise durations (right panel, *d*_*rise*_ < 22 s). As expected, the short-*d*_*rise*_ histogram exhibits many more SP responses and substantially fewer longer, more complex responses. This effect is compared with experimental data in section Variability between Astrocyte Subcompartments: Model.

#### Modeled response types are sensitive to the IP_3_ waveform

Figure 5B shows the total Ca^2+^ amount vs. the total IP_3_ amount (i.e., the areas under the Ca^2+^ and IP_3_ traces, respectively). Data points are color-coded by response type, and the symbol type denotes the IP_3_ rise durations (squares: *d*_*rise*_ < 22 s; circles: *d*_*rise*_ > 22 s, as described in section Materials and Methods). We draw several conclusions from Figure 5B and associated results. First, the total amounts of IP_3_ and Ca^2+^ are strongly positively correlated. In contrast, we found no correlation between other features of the IP_3_ and Ca^2+^ waveforms: IP_3_ rise duration, total duration, decay duration, amplitude, or the ratio of amplitude over duration did not correlate with Ca^2+^ total amount, duration, or amplitude. Second, although there is some correlation between total IP_3_ amount and Ca^2+^ response type, no two features or parameters of the IP_3_ waveform strictly predict the response type. Third, for intermediate values of total IP_3_ and Ca^2+^ amounts, the response type is particularly sensitive to small changes in the IP_3_ waveform (see the Figure 5B inset and example waveforms in Figure 5C; for more details on the model's sensitivity to various parameters, refer to Handy et al., 2017). This finding suggests that experimental trial to trial variability discussed earlier could be caused by small trial to trial changes in the IP_3_ waveform.

### Ca^2+^ fluxes as a source of response variability

**Figure 6:**
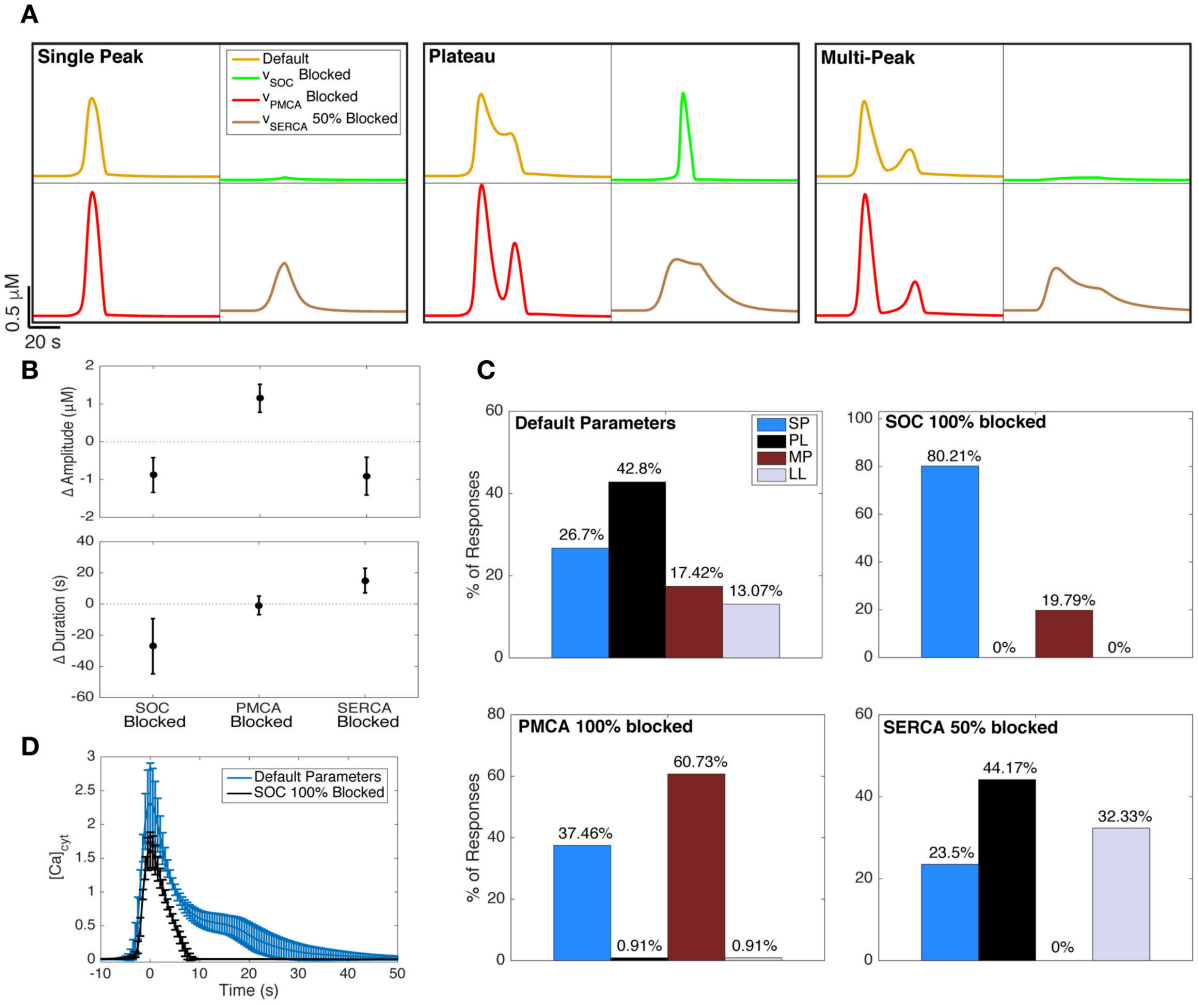
Effect of blocking Ca^2+^ channels and pumps on Ca^2+^ responses. **(A)** The effects of blocking SOC channels (green traces), PMCA pumps (red traces), and SERCA pumps (brown traces) on example SP, PL, and MP responses generated with the default model parameters (yellow traces). The underlying IP_3_ dynamics for each panel are, from left to right (in order, *A, d*_rise_, *r*_rise_, *d*_dec_): 0.2, 21, 0.002, 97 (SP), 0.375, 36, 0.002, 120 (PL), 0.2, 41, 0.15, 179 (MP). The IP_3_ input was applied 20 s after the start of the simulation. **(B)** The change in Ca^2+^ response amplitude and duration after blocking the three channels and pumps. For all 600 IP_3_ inputs, the mean change in response kinetics from the default Ca^2+^ response is shown, with its standard deviation. **(C)** The effects of blocking the channels and pumps on Ca^2+^ response type distributions. The upper left distribution (corresponding to default parameters) is repeated from the left panel of Figure 5A. **(D)** The average PL response with default model parameters (IP_3_ *d*_rise_ < 22 s, as in the right panel of Figure 5A) shown in blue. Using the same underlying IP_3_ dynamics as the input, we also generated the average Ca^2+^ response when SOC channels were fully blocked (black trace). As observed, the plateau phase of Ca^2+^ responses is eliminated when SOC channels are blocked in our model. To generate these average traces, the trace peaks were first aligned, ignoring latency. Error bars indicate standard deviations.

In addition to differences in IP_3_ dynamics, cell to cell and subcompartment to subcompartment response variabilities may also be due to different expression levels or functional properties of channels and pumps (e.g., SOC channels, PMCA pumps, and SERCA pumps) involved in Ca^2+^ responses (section Forms of Ca^2+^ Response Variability and their Sources). Therefore, we examined the individual contribution of each of these channels and pumps to Ca^2+^ responses (Figure 6). We also studied the effects of modifying Ca^2+^ leak fluxes between the cytosol and ER or ECS, and the volume ratio of the cytosol to the ER (γ), and found no major effects (Handy et al., 2017). In unpublished work, we have examined the effects of IP_3_R parameters including d_1_, the IP_3_ dissocation constant. Increasing d_1_ shifts to the right the bifurcation that gives rise to the complex transition to more complex and long-lasting responses in Figure 5B (data not shown), but does not lead to qualitative changes in behavior, and can be replicated by scaling IP_3_ and the parameter d_3_. Effects of the more complete set of parameters are considered in the context of bifurcation analysis (Handy et al., 2017).

#### Blocking SOC channels results mostly in SP responses

The green traces in Figure 6A show how the example Ca^2+^ responses with default parameters (yellow traces) change when SOC channels are fully blocked. As seen, the durations and amplitudes of the Ca^2+^ responses to the same IP_3_ input decrease when SOC channels are blocked. This was true for nearly 99% of the full set of 600 IP_3_ traces (summarized for complete set of responses in Figure 6B, left). In fact, for 120 out of the 600 choices for IP_3_ parameters, blocking SOC channels suppresses the response up to the degree that the response would potentially become undetectable in experiments (amplitude <0.4 μM). In other words, our model predicts that blocking SOC channels decreases the likelihood that the astrocyte will respond to a brief application of agonist.

Figure 6C (upper right histogram) illustrates that blocking SOC channels in the model increases the likelihood of observing SP responses, and completely eliminates the incidence of PL and LL responses. These data show that without functional SOC channels, we would expect to only observe SP or MP responses, at least during short-duration agonist applications. The fact that SOC channels have such strong effects on Ca^2+^ responses is particularly surprising given that the SOC flux rates are low in our model (Figure 3D; examined in more detail in Handy et al., 2017).

In agreement with our model prediction, previous experimental studies have shown that blocking astrocyte SOC channels eliminates the plateau phase of astrocyte Ca^2+^ responses and transforms these PL responses to SP responses (Malarkey et al., 2008; Pivneva et al., 2008; Wang et al., 2012). Figure 6D (blue trace) shows the average (± standard deviation) PL trace from the same set of model parameters as in the right panel of Figure 5A. Simulating responses using the same IP_3_ parameters, but with SOC channels fully blocked, eliminates the plateau phase of the responses, transforming them into SP responses (Figure 6D, black trace).

#### Blocking PMCA pumps increases the occurrence of MP responses

The red traces in Figure 6A show how the example SP, MP, and PL traces transform when PMCA pumps are fully blocked. As seen, the amplitudes, but not durations, of Ca^2+^ responses to the same IP_3_ input increase when PMCA pumps are fully blocked. The change in Ca^2+^ amplitude and duration, for any given IP_3_ input, arising when PMCA pumps are blocked is shown in Figure 6B, center. The response type distribution (Figure 6C, lower left) illustrates that blocking PMCA pumps almost entirely eliminates PL and LL responses (as was the case when blocking SOC channels). However, in contrast to the effects of blocking SOC channels, blocking PMCA pumps considerably increases the occurrence of MP responses.

#### Partial block of SERCA pumps eliminates MP responses

SERCA pumps are a necessary mechanism to fill the ER with Ca^2+^, and completely blocking these pumps experimentally has been shown to lead to apoptosis (Luciani et al., 2009). In our model, completely blocking this term would lead to the ER being entirely emptied, and the system would be unable to evoke a response with IP_3_. Therefore, we only investigated a 50% block of this channel. As seen in Figure 6A (brown traces), the durations of Ca^2+^ responses increase when SERCA pumps are partially blocked, while the amplitudes decrease. This change in Ca^2+^ kinetics occurred for all 600 IP_3_ traces and is summarized in Figure 6B, right. Furthermore, the response type distribution (Figure 6C, lower right) shows that MP responses are entirely eliminated when SERCA pumps are 50% blocked, and mostly transform into additional LL responses.

### Variability between astrocyte subcompartments: model

Thus, far, we have examined the separate effects of IP_3_ dynamics and individual Ca^2+^ fluxes on resulting Ca^2+^ dynamics. We now consider the effects of simultaneously changing both IP_3_ parameters and Ca^2+^ channel/pump fluxes within a biologically plausible range (Table 1), in order to explore how these differences may shape the variety of response type distributions among the somas, large processes, and small processes (Figure 2D). To do this, we ran simulations with random combinations of the three flux parameters *v*_SOC_, *v*_PMCA_, and *v*_SERCA_, while drawing the IP_3_ *d*_rise_ parameter from different ranges (for details see section Materials and Methods). We then selected the subspace, or subspaces, that best matched each of the three experimentally-recorded distributions in Figure 2D. A more detailed examination of the response type distributions resulting from each of these subspaces is provided in Handy et al. (2017).

The rationale behind this parameter search is that Ca^2+^ response variability between astrocyte subcompartments stems from variability in both Ca^2+^ channel/pump properties and the underlying IP_3_ kinetics (see sections Variability between Astrocyte Subcompartments and Contribution of IP3 to Ca^2+^ Response Variability). It is noteworthy that the simulations for a given astrocyte subcompartment also include a range of Ca^2+^ channel/pump parameters and IP_3_ parameters. Since in the experiments multiple cells were used to generate the data for each astrocyte subcompartment, having a range of parameters in simulations reflects the variability in channel/pump properties and IP_3_ kinetics inherent to each cell (see section Trial to Trial and Cell to Cell Variability on cell to cell variability). The range of IP_3_ parameters in simulations for each subcompartment also reflects experimental IP_3_ stochasticity stemming from trial to trial variability (i.e., the same ROI in the same cell responding differently to identical agonist pulses; see sections Trial to Trial and Cell to Cell Variability and Contribution of IP3 to Ca^2+^ Response Variability) as well as experimental variability (e.g., pipette distance from the ROIs).

**Figure 7:**
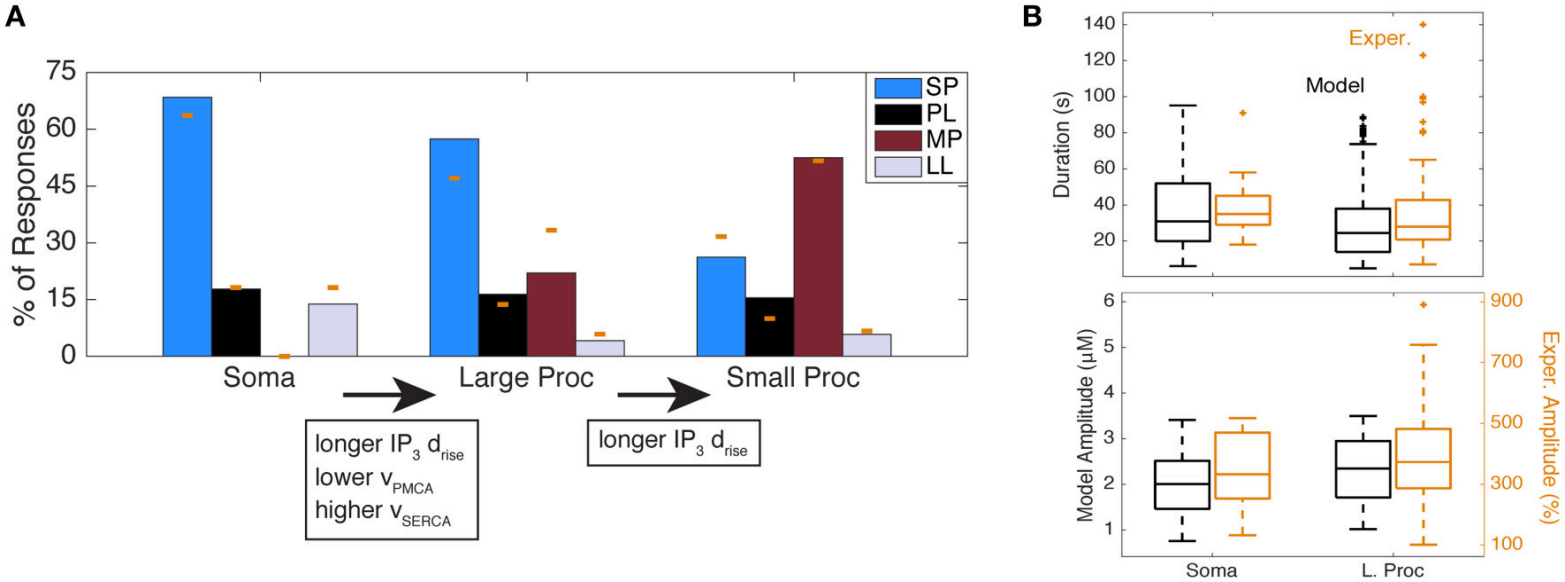
Simulation of response type distributions matching experimental distributions of astrocyte subcompartments. **(A)** Response type distributions in the model with parameters adjusted to the soma, large processes, and small processes. The yellow bars indicate the experimental data from Figure 2D. **(B)** Ca^2+^ durations and amplitudes of all response types in the model (from **(A)**; in black) and experiments (from Figure 2D; in yellow) in the soma and large processes. One outlier was excluded from the experimental soma duration plot, with a duration of 363 s (same outlier omitted from Figure 4B).

Using this parameter search, we found that the only subspace with a distribution that closely resembled the somatic distribution consisted of very short IP_3_ *d*_rise_ values (≤11 s) and the following parameter ranges: high *v*_SOC_ (1.83–2.36), medium *v*_PMCA_ (8.33–11.67), and low *v*_SERCA_ (0.45–0.75). We did not find any one subspace that matched the distributions of the large and small processes. By looking at combinations of two adjacent subspaces, we found that IP_3_ *d*_rise_ values ≤21 s and the following parameter ranges provided a distribution similar to that of the large processes: high *v*_SOC_ (1.83–2.36), low *v*_PMCA_ (5.0–8.33), and medium and high *v*_SERCA_ (0.75–1.35). Using this same parameter subspace of the large processes, but with the full range of IP_3_ *d*_rise_ values in Table 1 (≤41 s), we obtained a distribution similar to that of the small processes. The three Ca^2+^ response type distributions from the mathematical model are shown in Figure 7A. The following are the percentages of observed response types in these random simulations (in order, SP, PL, MP, LL) for each subcompartment: 68.42, 17.76, 0.0, 13.82% (soma); 57.44, 16.37, 22.02, 4.17% (large processes); 26.21, 15.46, 52.52, 5.81% (small processes).

To confirm that the Ca^2+^ response kinetics generated from these parameter subspaces are reasonable and comparable to the kinetics in the experimental data, we examined the ranges of Ca^2+^ kinetics for the soma and large processes of all response types in experiments (from the histogram in Figure 2D) and the random model simulations. We found that, similar to experiments, the model Ca^2+^ responses consist of a wide range of Ca^2+^ durations and amplitudes (Figure 7B), which is expected given the trial to trial and cell to cell variability we had discussed previously (Figure 2C). Moreover, while exact amplitude values between the model and experiments are not comparable (due to different measurement units), we see that response durations are very similar.

In summary, our results predict that the main differences between the astrocyte somas and large processes that are responsible for different response type distributions are as follows: (1) large processes include IP_3_ dynamics with slightly longer rise durations, and (2) the Ca^2+^ flux rates through the PMCA and SERCA pumps are, respectively, lower and higher in the large processes. Response type distributions associated with small processes can be generated by increasing IP_3_ rise durations even further, but using the same Ca^2+^ flux parameters as for large processes. This gradient in IP_3_ rise durations from somas to fine processes could be related to the influx of IP_3_ through spatial diffusion from other subcompartments, the constricted volume of the smaller subcompartments compared to the larger ones, and different probabilities of the agonist binding to receptors (due to the different subcompartment sizes and GPCR densities or expression levels).

## Discussion

We analyzed astrocyte Ca^2+^ responses to brief focal ATP applications and, based on our results, developed a single-compartment model of astrocyte Ca^2+^ activity to investigate mechanisms of Ca^2+^ response variability. We categorized both experimentally-recorded and model Ca^2+^ responses into four types: Single-Peak (SP), Multi-Peak (MP), Plateau (PL), and Long-Lasting (LL). We found that, experimentally, SP response kinetics do not differ consistently between the soma, and processes, but we do see variability between cells and trials. However, the distributions of Ca^2+^ response types vary between different astrocyte subcompartments, with the likelihood of SP responses decreasing, and of MP responses increasing, in the large and small processes relative to the soma.

Our model responses were tuned to match the average experimental short-duration SP response kinetics. We then applied IP_3_ transients to the model with a range of biologically plausible kinetics, and found that this range of IP_3_ parameters was sufficient to reproduce all four Ca^2+^ response types in the model, without the need for feedback-induced oscillations in the IP_3_ waveform (Politi et al., 2006). The model was also used to predict how blocking astrocyte Ca^2+^ channels and pumps would affect the Ca^2+^ responses. In particular, blocking SOC channels resulted mostly in SP responses, and decreased the response duration and amplitude. Blocking PMCA pumps eliminated most PL and LL responses, and increased response amplitude. Finally, blocking SERCA pumps eliminated MP responses, decreased response amplitude, and increased the duration.

Lastly, we propose that the experimentally observed response variability between astrocyte subcompartments can be explained by the differences in their channel/pump flux rates and IP_3_ kinetics. Namely, our simulations suggest that: (1) astrocyte somas have higher PMCA flux rates and lower SERCA flux rates than the processes, and (2) when moving from the somas to the large and small processes, the underlying IP_3_ transients tend to include those with longer rise durations.

Our modeling results highlight two major advantages of relatively simple, biophysically based, mathematical models. First, they are experimentally falsifiable, and thus can be straightforwardly improved, or if necessary, discarded. Second, verified models of this type can be used as diagnostic tools. For example, because our model makes strong predictions about how specific biophysical mechanisms determine the types of Ca^2+^ transients evoked in astrocytes, it is potentially very useful in determining which mechanisms are altered in disease states, reactive astrocytosis, or other cases in which Ca^2+^ events are potentially altered.

### Contribution of IP_3_ to Ca^2+^ response variability

We have presented three contexts in which astrocyte response variability has been observed (Table 2). Our results suggest that IP_3_ time course variability may be a key contributor to all forms of response variability. For a given agonist application, variability between cells, or subcompartments within a cell, may arise from the profile of agonist reaching the GPCRs (e.g., due to different subcompartment sizes/shapes, or distances from the pipette); the local expression level or properties of GPCRs in a given cell or subcompartment; and differences in the diffusion and degradation of IP_3_. Our experimental and computational results suggest that the net effect of such differences between the soma, large processes, and small processes is to increase the effective IP_3_ rise duration (*d*_*rise*_) in the periphery relative to the soma (Figure 7A). In our model, further differences in PMCA and SERCA flux rates (*v*_PMCA_, *v*_SERCA_) are necessary to explain differences between the distributions of responses between somas and processes.

For a given ROI, we see much more trial to trial response variability than in IP_3_ uncaging experiments (Fiacco and McCarthy, 2004). In contrast with Toivari et al. (2011), we thus conclude that the dominant source of trial to trial variability lies in the factors that determine the IP_3_ waveform in response to repeated agonist applications. IP_3_ waveform variability and concomitant variability in Ca^2+^ responses has been observed directly in response to bath application of ATP in other cell types (Tanimura et al., 2009; Nezu et al., 2010).

What is the source of this hypothesized trial to trial variability in the IP_3_ waveform? Because we see no obvious trends in response to repeated stimuli, we speculate that the dominant factor is stochasticity from two sources. First, the number of activated GPCRs may vary stochastically between trials. Second, the mechanisms governing IP_3_ dynamics downstream of GPCRs are sensitive to varying molecule numbers known to assist with IP_3_ metabolism (Bartlett et al., 2015), and can lead to robust changes in Ca^2+^ signals. Another well-described source of stochasticity in Ca^2+^ responses is the inherent stochasticity of the IP_3_R (Falcke, 2003; Dupont and Sneyd, 2009; Dupont et al., 2016) and other Ca^2+^ channels (Skupin et al., 2008, 2010). However, we believe that stochastic gating of these channels is less dominant for ATP-pulse-evoked Ca^2+^ transients studied here than for the spontaneous events, due to the larger number of IP_3_ molecules involved. Our view is supported by Fiacco and McCarthy (2004), who uncaged IP_3_, thus presumably reducing variability in the IP_3_ waveform. They saw much less variability in evoked Ca^2+^ events compared with our ATP protocol, suggesting that the dominant sources of variability are upstream from the IP_3_ waveform for evoked transients. Taken together, the body of experimental and modeling results thus suggests that sources of variability are quite different for spontaneous and evoked events. We consider all the potential sources of stochasticity described here ripe for future study.

### Ca^2+^ oscillations and plasma membrane fluxes

Our modeling results suggest that the presence of SOC channels allows for sustained Ca^2+^ oscillations without oscillations in IP_3_, despite the low SOC flux rates (Figure 3D). This result is consistent with experimental results from astrocytes (Sergeeva et al., 2000, 2003). We explore this issue in more detail in Handy et al. (2017). A similar role may be played by receptor-operated Ca^2+^ (ROC) channels, which are activated by GPCR agonists rather than by the depleted ER. ROC channels have been found in some astrocytes (Grimaldi et al., 2003; Beskina et al., 2007) and included in earlier models (Croisier et al., 2013). Regardless of the mechanism for plasma membrane Ca^2+^ fluxes, our model suggests the importance of considering an open-cell model in which total intracellular Ca^2+^ levels are allowed to fluctuate.

### Comparison of experimental and model Ca^2+^ response kinetics

Although our model accounts very well for the variety of measured Ca^2+^ transients (Figures 4, 7B), it appears unable to account for a handful of very long (duration >120 s), often oscillatory events. Like nearly any tractable mathematical model, our model was not designed to account for all possibilities that may have been encountered in the experiments. Several factors, not accounted for in the model but possible in the experiments, could contribute positive feedback to extend Ca^2+^ transients in rare cases. First, applied ATP may cause release of other GPCR agonists from nearby glia or neurons, thus extending the duration of GPCR activation (Anderson et al., 2004). Second, astrocytes produce spontaneous Ca^2+^ activity via mechanisms that are incompletely understood but distinguishable from those implicated in evoked transients (Aguado et al., 2002; Wang et al., 2006; Haustein et al., 2014; Srinivasan et al., 2015). ATP application or evoked transients may interact with this mechanism to prolong the Ca^2+^ response in rare cases. Lastly, although single IP_3_ pulses can drive Ca^2+^ oscillations in our model, it is not known whether astrocytes generate multiple pulses or oscillations in IP_3_ levels in response to ATP pulses, as is seen, e.g., in HYS-EA1 cells during agonist bath applications of ATP (Tanimura et al., 2009). Feedback from Ca^2+^ to IP_3_, which has not been demonstrated in astrocytes except in tissue cultures, can in principle generate IP_3_ oscillations that enhance Ca^2+^ oscillations and prolong their duration (Höfer et al., 2002; Politi et al., 2006).

Similar mechanisms may underlie rare, experimentally observed Ca^2+^ oscillations with progressively increasing peak heights (e.g., the second MP and PL examples in Figure 2A), which are only reproducible in our model with more than one IP_3_ pulse (data not shown). Consistent with the suggestion that oscillatory IP_3_ events underlie growing Ca^2+^ oscillations, increases in Ca^2+^ peaks have been observed in HSY-EA1 cells during IP_3_ oscillations (Tanimura et al., 2009, Figure 5). Moreover, in COS-7 cells where IP_3_ did not oscillate, Ca^2+^ oscillations were possible but the initial Ca^2+^ peak was the largest peak for any given agonist concentration (Tanimura et al., 2009).

Several known Ca^2+^ buffering and exchange mechanisms, including the Na^+^/Ca^2+^-exchanger and the effects of mitochondria, were not explicitly included in our model. We left these mechanisms out for three reasons. First, we simply lack adequate data from astrocytes to build such a model in good faith. Second, our minimal model was adequate to describe the experimental data sets quantitatively, with the exception of rare, long-lasting events. Third, our model is simple enough to allow formal mathematical analysis (Handy et al., 2017) that gives a significantly deeper understanding of the model's behavior. Development of a more mechanistically complex and detailed model awaits more detailed data, collected from astrocytes in the presence of appropriate blockers, so that the effects of each buffering component can be studied in isolation.

### Role of astrocyte Ca^2+^ responses

Ca^2+^ transients have been hypothesized to have diverse downstream effects, including multiple forms of gliotransmission, modulation of transporters, and gene expression (reviewed by Bazargani and Attwell, 2016). It is likely that transients generated in different subcompartments generate different outcomes. For example, due to peripheral processes' proximity to neuronal synapses and blood vessels, small processes may be more involved in regulation of synaptic function and blood flow. The different response type distributions of each astrocyte subcompartment (Figure 2D), may be a reflection of their different roles.

Much of our analysis has focused on dividing Ca^2+^ responses into one of four types. This approach can be a useful tool for researchers, because the distribution of response types is clearly related to the underlying biophysics. However, it is unlikely that astrocytes encode information based solely on Ca^2+^ response type, since small changes in IP_3_ time course may change response types (Figure 5C). Instead, our results suggest that the most controllable way to reliably “encode” for different outcomes (Bazargani and Attwell, 2016) is via total Ca^2+^ amount (related to response type, duration, and amplitude), which varies more smoothly with small differences in the upstream triggering events (Figure 5B).

## Author contributions

MT collected and analyzed the experimental data under the guidance of JW. MT and GH built the mathematical model, ran the simulations, and analyzed the results under the guidance of AB. AB and JW oversaw the design and execution of project. MT, GH, AB, and JW wrote and reviewed the manuscript.

### Conflict of interest statement

The authors declare that the research was conducted in the absence of any commercial or financial relationships that could be construed as a potential conflict of interest.
