# Supplementary material for: Diversity of Evoked Astrocyte Ca2+ Dynamics Quantified through Experimental Measurements and Mathematical Modeling
Source: Front Syst Neurosci. 2017 Oct 23;11:79. doi: 10.3389/fnsys.2017.00079 (PMC5660282; doi:10.3389/fnsys.2017.00079)
Supplement: Supplementary file 1 [file Image1.PDF]

Supplementary Material

Diversity of Evoked Astrocyte Ca^{2+} Dynamics Quantified Through Experimental Measurements and Mathematical Modeling

Marsa Taheri[+], Gregory Handy[+], Alla Borisyuk[*++], John A. White[*++]

+: Co-first authors. ++: Co-corresponding authors

*** Correspondence:** Alla Borisyuk: borisyuk@math.utah.edu, John A. White: jwhite@bu.edu

1 Supplementary Figure

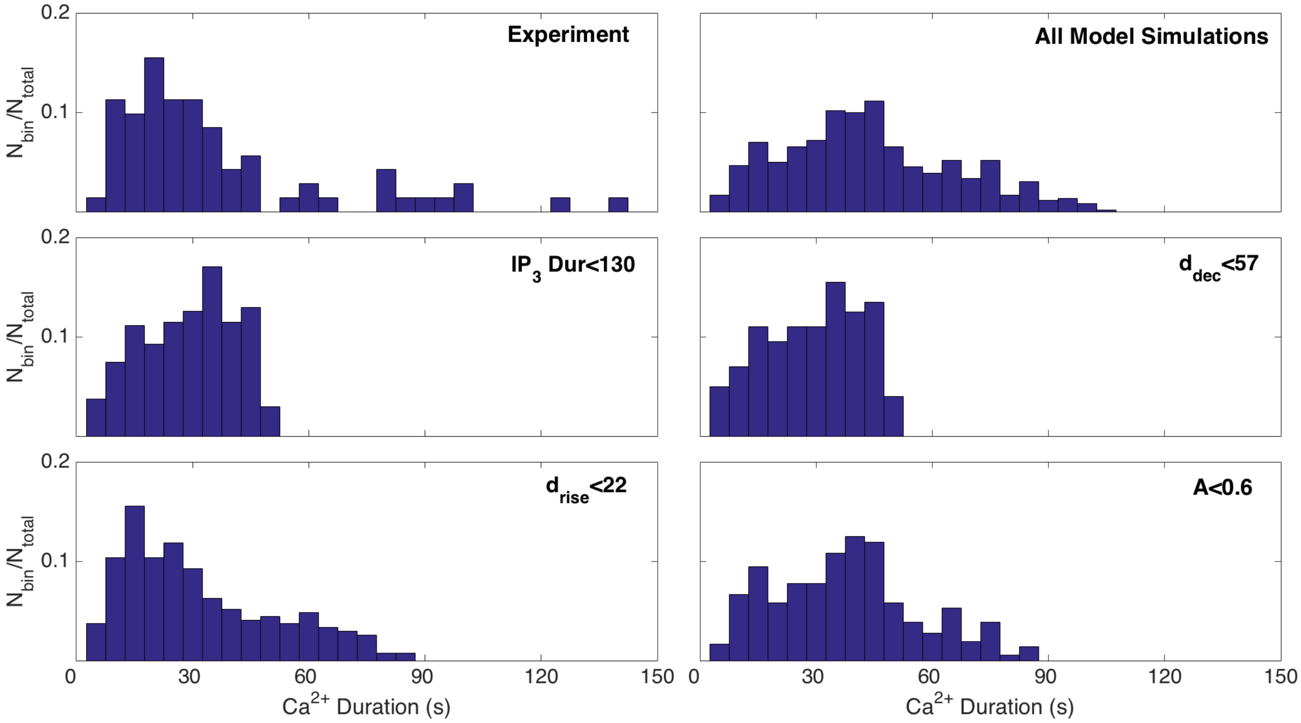

Supplementary Figure 1. Distribution of experimental and model Ca^{2+} transient durations. The distribution of Ca^{2+} response durations from experimental data (top left; same data as in Fig. 4B, with the same outlier omitted) was significantly different from the distribution of model Ca^{2+} transients (top right; same data as in Fig. 4A). Limiting the IP_3 parameters in the model (total IP_3 duration, IP_3 decay duration, IP_3 rise duration, and IP_3 amplitude) changes the distribution of Ca^{2+} transient durations as shown. The only distribution that is not significantly different from the distribution of experimental Ca^{2+} durations is the one where IP_3 rise durations are limited to less than 22 s (bottom left).
